# MLG-YOLO: A Model for Real-Time Accurate Detection and Localization of Winter Jujube in Complex Structured Orchard Environments

**DOI:** 10.34133/plantphenomics.0258

**Published:** 2024-09-23

**Authors:** Chenhao Yu, Xiaoyi Shi, Wenkai Luo, Junzhe Feng, Zhouzhou Zheng, Ayanori Yorozu, Yaohua Hu, Jiapan Guo

**Affiliations:** ^1^ College of Optical, Mechanical and Electrical Engineering, Zhejiang A&F University, Hangzhou 311300, China.; ^2^ College of Mechanical and Electronic Engineering, Northwest A&F University, Yangling 712100, China.; ^3^ Institute of Systems and Information Engineering, University of Tsukuba, Tsukuba 305-8573, Japan.; ^4^ Bernoulli Institute for Mathematics, Computer Science and Artificial Intelligence, University of Groningen, 9747 AG Groningen, The Netherlands.

## Abstract

Our research focuses on winter jujube trees and is conducted in a greenhouse environment in a structured orchard to effectively control various growth conditions. The development of a robotic system for winter jujube harvesting is crucial for achieving mechanized harvesting. Harvesting winter jujubes efficiently requires accurate detection and location. To address this issue, we proposed a winter jujube detection and localization method based on the MobileVit-Large selective kernel-GSConv-YOLO (MLG-YOLO) model. First, a winter jujube dataset is constructed to comprise various scenarios of lighting conditions and leaf obstructions to train the model. Subsequently, the MLG-YOLO model based on YOLOv8n is proposed, with improvements including the incorporation of MobileViT to reconstruct the backbone and keep the model more lightweight. The neck is enhanced with LSKblock to capture broader contextual information, and the lightweight convolutional technology GSConv is introduced to further improve the detection accuracy. Finally, a 3-dimensional localization method combining MLG-YOLO with RGB-D cameras is proposed. Through ablation studies, comparative experiments, 3-dimensional localization error tests, and full-scale tree detection tests in laboratory environments and structured orchard environments, the effectiveness of the MLG-YOLO model in detecting and locating winter jujubes is confirmed. With MLG-YOLO, the mAP increases by 3.50%, while the number of parameters is reduced by 61.03% in comparison with the baseline YOLOv8n model. Compared with mainstream object detection models, MLG-YOLO excels in both detection accuracy and model size, with a mAP of 92.70%, a precision of 86.80%, a recall of 84.50%, and a model size of only 2.52 MB. The average detection accuracy in the laboratory environmental testing of winter jujube reached 100%, and the structured orchard environmental accuracy reached 92.82%. The absolute positioning errors in the *X*, *Y*, and *Z* directions are 4.20, 4.70, and 3.90 mm, respectively. This method enables accurate detection and localization of winter jujubes, providing technical support for winter jujube harvesting robots.

## Introduction

Winter jujube is a popular and nutritious fresh fruit in China [1] and has traditionally relied on manual harvesting, a process that is time consuming, labor intensive, and costly [2]. Recently, with the increase in structured winter jujube orchards, robotic harvesting has become a progressive trend. The key task of harvesting robots is to utilize visual sensing for crop information perception [3], including fruit detection and location, to plan harvesting strategies [4]. However, robotically harvested winter jujubes are limited by their small size and frequent obstructions to branches and leaves, which reduces the robots’ recognition accuracy [5]. Therefore, the precise detection and localization of winter jujubes have emerged as critical components of the robotic harvesting process.

The advent of deep learning has been a key development in image processing for several years [6]. This has provided new solutions for object recognition and location in robotic harvesting [7]. The main deep learning-based object detection algorithms include Fast R-CNN [8], Mask R-CNN [9], R-FCN [10], SSD [11], and YOLO [12,13]. Among them, Faster R-CNN, Mask R-CNN, and R-FCN are 2-stage algorithms that use region extraction and convolutional neural network feature processing for detection, which is slower. Single-stage algorithms such as SSD and YOLO do not require generating candidate regions or priors and output the location and category of the object directly through an end-to-end neural network, making the detection process more real time and lightweight. These algorithms have been widely used in the recognition of various fruits, such as kiwi [14], apple [15], orange [16], mango [17], grape [18], and lychee [19]. In terms of jujube recognition, Xu et al. [20] proposed a method called “YOLO-Jujube” that can automatically detect jujube fruits and their ripeness in a natural environment. Tianzhen et al. [21] proposed a YOLO v3-SE model based on YOLO v3 and embedded it with SE Net to strengthen important and effective features, thereby improving the performance of feature maps. The improved YOLOv5s model proposed by Feng et al. [22] replaces the neck of YOLOv5s with a slim neck, which reduces the network complexity and improves the performance, achieving an accuracy of 88.70% in detecting winter jujubes. Zheng et al. [23] proposed a winter jujube recognition model using an improved YOLOX-Nano, which optimized the training process with DIoU loss and achieved a lightweight model with a size of only 4.47 MB.

The main current methods for fruit positioning include laser scanning technology, stereo vision systems, and RGB-D camera-based methods. For example, Tsoulias et al. [24] used a LiDAR laser scanner to detect and locate apples in orchards, with an average detection success rate of 92.50% for defoliated trees, demonstrating the potential of remote apple detection and 3-dimensional (3D) positioning. Hou et al. [25] used an improved YOLOv5s and binocular vision method to accurately detect and locate mature citrus fruits in orchards. Jianjun et al. [26] proposed a binocular system measurement model based on neural networks for 3D positioning of tomatoes, achieving a positioning reliability of 88.58% when the error limits in the X, Y, and Z results were within 5 mm. However, laser scanning technology and stereo vision systems also have limitations. For example, laser scanning technology is costly and may not adequately recognize small or partially obscured fruits [27]. Stereo vision systems can provide spatial depth information but may encounter difficulties in processing occluded objects [28]. Fu et al. [29] emphasized the potential of RGB-D cameras in the field of fruit detection and positioning. RGB-D cameras combine color images and depth information to accurately detect and position fruits under different lighting conditions and complex backgrounds while offering marked advantages in terms of cost-effectiveness, flexibility, and adaptability. Arad et al. [30] proposed a robotic system for harvesting sweet peppers using an eye-in-hand positioning scheme and combining a depth camera at the end of a robotic arm for 3D positioning of sweet peppers. Li et al. [31] developed a fruit load branch algorithm based on an RGB-D camera for simultaneously detecting and positioning multiple lychee fruit clusters in a large environment by combining the semantic segmentation method DeepLabv3 and binary image processing methods. Additionally, when combined with deep learning algorithms such as YOLO for object detection, RGB-D cameras can achieve efficient and accurate fruit positioning, meeting the needs of real-time processing. Li et al. [32] improved the YOLOv5 model for citrus recognition and used an RGB-D camera for citrus positioning. Hu et al. [33] proposed an improvement of the YOLOX model for detecting fruit regions in object images, with an RGB-D camera obtaining aligned depth images to determine the coordinates of apple-picking points.

This study aims to solve this problem in robotic winter jujube harvesting by designing an efficient and lightweight object detection algorithm and further combining RGB images with depth information obtained from RGB-D cameras for the winter jujube 3D positioning. The main contents of this study are as follows:

1. A winter jujube object detection model, MLG-YOLO, is proposed to incorporate MobileViT to reconstruct the backbone of YOLOv8n, reducing computational complexity and making the model more lightweight.

2. The LSKblock and GSConv modules are introduced in the neck module of YOLOv8n. Through its spatial selection mechanism and dynamic adjustment of large kernels, LSKblock can effectively handle the context at different spatial positions in object detection. The GSConv module simplifies feature fusion through mixed operations. By combining the 2 modules, the model can detect small winter jujubes more accurately.

3. A 3D localization method is proposed for winter jujubes by combining MLG-YOLO with RGB-D cameras, and experiments are designed to evaluate localization errors. The methods are shown to improve the accuracy of jujube detection and localization in complex orchard environments and meet the requirements of robotic harvesting systems.

## Materials and Methods

### Image acquisition and preprocessing

**Fig. 1.**
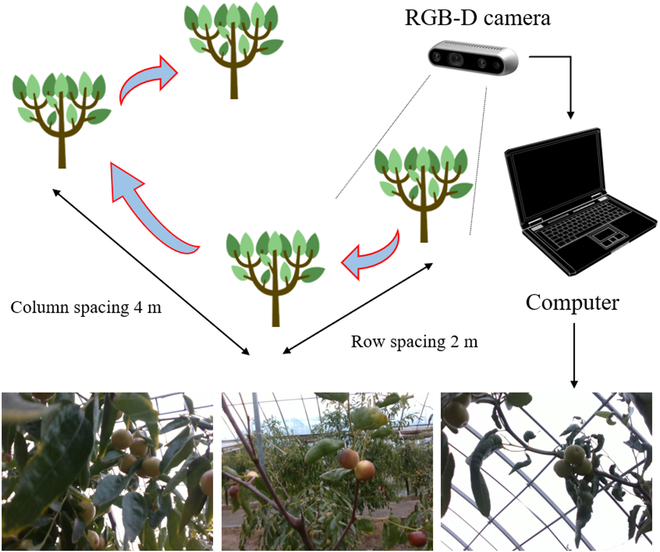
Schematic diagram of winter jujube dataset acquisition.

The images for this study were acquired at the winter jujube experiment and demonstrate station of Northwest Agriculture and Forestry University. These winter jujube images were captured with an RGB-D camera at 640 × 480 resolutions. The dataset was collected during 3 different periods of the day under various weather conditions, resulting in a total of 930 winter jujube images, as illustrated in Fig. 1.

These 930 images were randomly partitioned into a training, validation, and test set in the ratio of 8:1:1. To enhance the diversity and robustness of the dataset, a variety of image processing techniques were used, as changes in brightness, translation, mirroring, and rotation, thus tripling the dataset. Consequently, the final dataset comprised 2,232 training images, 279 validation images, and 279 test images. Additionally, all images were precisely annotated using LabelImgV1.8.6 software.

### Winter jujube detection based on MLG-YOLO

#### YOLOv8n model

YOLOv8 is Ultralytics’ latest object detection algorithm proposed in 2023, which builds on the success of the established YOLO series of algorithms by incorporating new features and improvements that greatly enhance performance and flexibility. The YOLOv8 network model consists of 3 main components: backbone, neck, and head. The backbone, responsible for feature extraction from images, has transitioned from the C3 structure in YOLOv5 to the C2f structure, offering richer gradient flow and enhanced feature extraction efficiency. The neck, which further processes these features, integrates the feature pyramid network [34] and path aggregation network [35] structures from predecessors such as YOLOv7 [36], allowing the model to extract useful semantic information from deeper layers and precise spatial information from shallower layers. The head, which handles the final object detection task, adopts a decoupled head structure and transitions from anchor based to anchor free. YOLOv8 offers various models for different application scenarios, with YOLOv8n being the lightest in terms of parameters and floating-point operations (FLOPs), meeting the needs of lightweight deployment. This study chooses YOLOv8n as the baseline model for improvement, aiming to enhance the detection accuracy for winter jujubes and achieve real-time detection.

#### MLG-YOLO model

In the orchard environment, detecting winter jujubes is limited by various natural conditions, particularly differences in lighting conditions. Changes in sunlight intensity and angle at different times can significantly alter image brightness and contrast. Moreover, the complex orchard background, such as leaves, branches, and the ground, and the obstruction of winter jujubes by branches and leaves increase the difficulty of object detection.

**Fig. 2.**
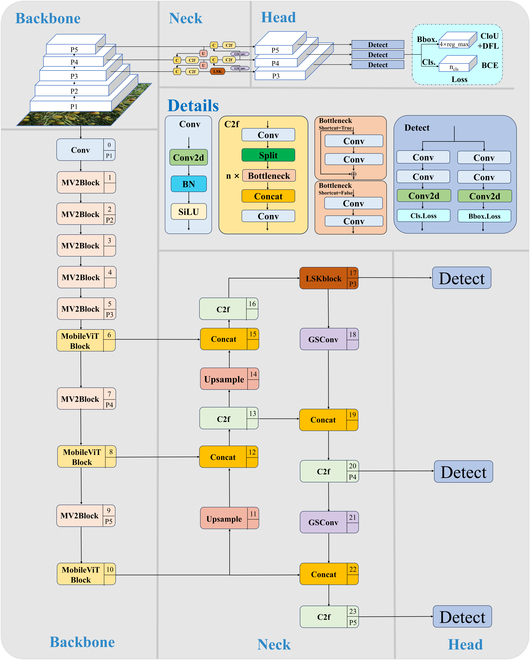
The architecture of the MLG-YOLO model.

This study proposes an MLG-YOLO model for detection of winter jujubes in natural environments to address these challenges effectively. This model improves upon the YOLOv8n model in 3 main aspects: incorporating MobileViT to reconstruct the YOLOv8n backbone, introducing the LSKblock (large selective kernel block) in the neck for enhanced detection capabilities in varying scales and complex backgrounds, and introducing a new lightweight convolutional technology, GSConv, to reduce the computational load while maintaining detection accuracy. The architecture of the MLG-YOLO model is shown in Fig. 2, and the specifics of these improvements will be further detailed.

#### Lightweight vision transformer: MobileViT

Since the introduction of vision transformers (ViTs) in 2020, they have shown impressive expressiveness and transferability in computer vision [37]. However, compared with lightweight, easily optimized convolutional neural networks (CNNs), ViTs are heavyweight. They lack spatial inductive bias and are more challenging to optimize, requiring more training data, iterations, data augmentation, and larger regularization terms.

**Fig. 3.**
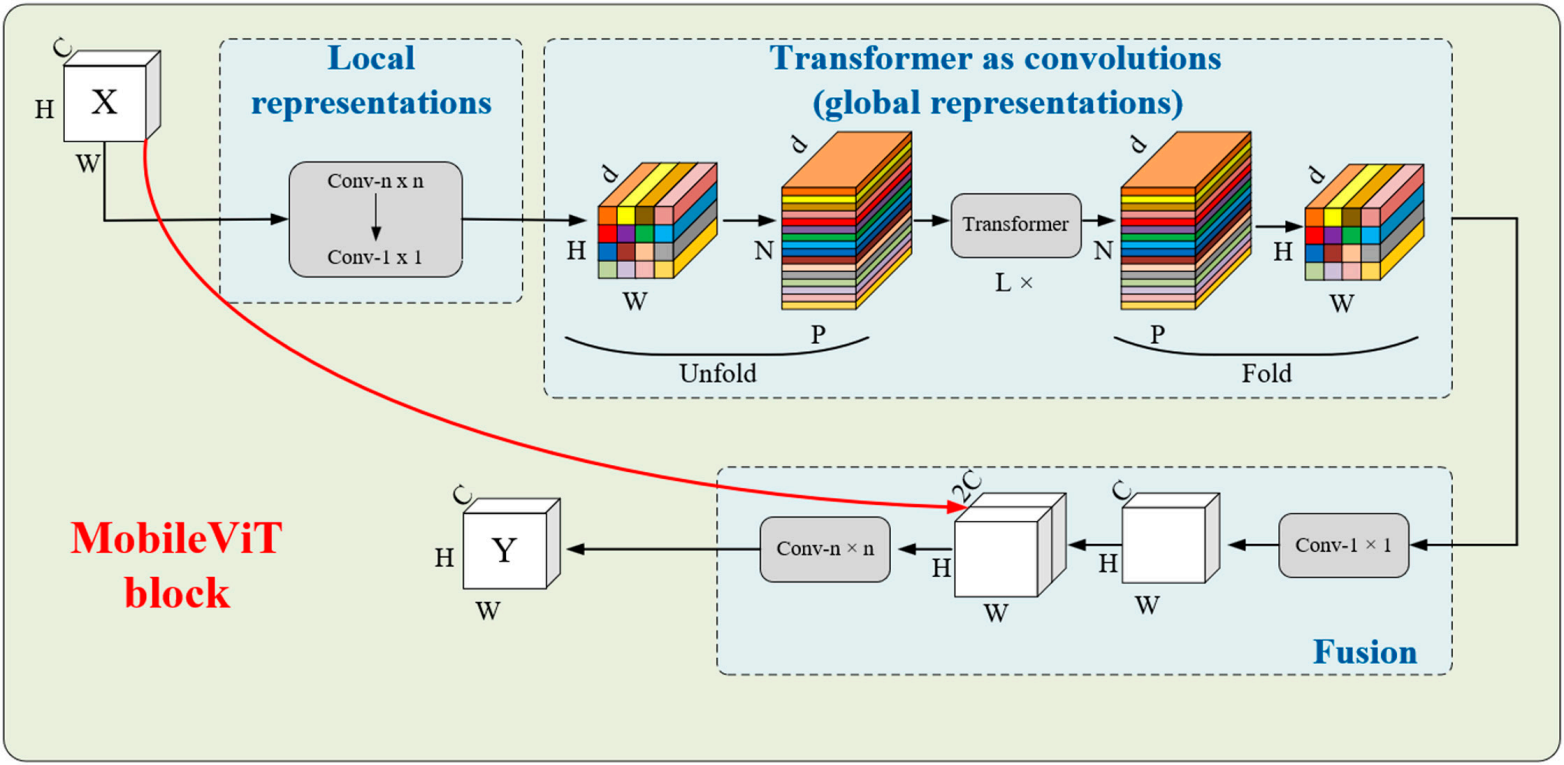
The architecture of the MobileViT block.

In contrast to standard ViTs, the hybrid architecture of CNNs with transformers offers advantages. CNNs provide spatial inductive bias, mitigating positional bias, and their integration accelerates network convergence, stabilizing the training process. This study employs the lightweight, general-purpose network MobileViT [38], which combines the benefits of convolutions and transformers to reconstruct the backbone of YOLOv8n. In contrast to standard ViTs, MobileViT adopts a novel method for learning global representations. It utilizes transformers for global information processing, contrary to the localized processing in conventional convolutions. This strategy facilitates more efficient representation learning, demanding fewer parameters and reduced data augmentation. The architecture of the MobileViT block is shown in Fig. 3.

The MobileViT block, as depicted in Fig. 3, is designed to capture both local and global information in an input tensor using a more efficient parameter setup. In essence, given an input tensor *X* with dimensions *H* × *W* × *C*, MobileViT applies a standard convolutional layer of size *n* × *n*, followed by a pointwise (1 × 1) convolutional layer, resulting in a transformed tensor *X_L_* with dimensions *H* × *W* × *d*, where *d* is a higher-dimensional space (*d* > *C*). The *n* × *n* convolutional layer encodes local spatial information, while the pointwise convolutional layer accomplishes this by learning linear combinations of the input channels.

To further enhance the capacity of MobileViT for global representation, *X_L_* is unfolded into N separate flattened patches, represented as X_U_ with dimensions *P* × *N* × *d*, where *P* is determined by the formula *P* = *wh* and *N* is calculated as *N* = *HW*/*P*, indicating the total number of these patches. *h* and *w* denote the height and width of each patch, respectively. *h* and *w* are both less than or equal to *n*. For each patch, denoted as *p* within the set of patches from 1 to *P*, we establish interpatch connections by applying transformers, resulting in the generation of *X_G_* with dimensions *P* × *N* × *d* as follows:

$$X_{G}\left(p\right)=T\text{ransformer}\left(X_{U}\left(p\right)\right),1\leq p\leq P$$ (1)

In contrast to ViTs, which can lose the spatial order of pixels, MobileViT retains both the order of patches and the spatial arrangement of pixels within each patch. Consequently, this works by folding an *X_G_* of dimension *P* × *N* × *d* into an *X_F_* of dimension *H* × *W* × *d*. Then, we transform *X_F_* into a lower-dimensional space with *C* dimensions using a pointwise convolution and combine it with *X* through concatenation. Subsequently, another *n* × *n* convolutional layer is applied to fuse these concatenated features.

#### Large selective kernel network

**Fig. 4.**
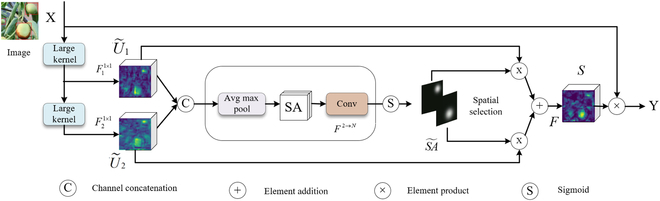
The architecture of the LSK module.

This study integrates the large selective kernel network (LSKNet) into the YOLOv8n framework [39], specifically in terms of the network’s neck output, to address the challenge of accurately detecting winter jujubes in complex orchard environments. The LSKNet’s design, which incorporates large, deep convolutional kernels with a spatially selective mechanism, dynamically adjusts the receptive field, catering to the diverse and variable contexts of the objects. This is crucial for minimizing the interference of branches and leaves in fruit recognition. The LSK module, depicted in Fig. 4, employs large kernel convolutions alongside a spatial kernel selection mechanism, demonstrating its ability to adaptively accumulate extensive receptive fields for varying objects.

Large kernel convolutions: By decomposing the convolution process into a series of depthwise convolutions, the model achieves larger kernel convolutions. This decomposition features progressively increasing kernel sizes and dilation rates, facilitating a rapid expansion of the receptive field and enabling the model to extract features with varied contextual information from different segments of the input.

Spatial kernel selection: The network employs a spatial selection mechanism that focuses on the spatial contexts most relevant to object detection. This mechanism involves selecting feature mappings from large convolutional kernels of various scales, enhancing the network’s ability to capture spatial relationships. It employs channel-based average and maximum pooling to process these spatial features, subsequently transforming them into spatial attention maps through a convolution layer. These maps, generated using a sigmoid activation function, create individual spatial selection masks for each large kernel. The model combines features from these kernels, weighted by their respective spatial selection masks, to produce attention features that are then elementwise multiplied with the input features to yield the LSK module’s final output.

where *X* represents the input feature, *F*^1×1^ is the 1 × 1 convolution layer, *SA* denotes the pooled spatial feature descriptors, $\tilde{\mathrm{SA}}$ denotes an individual spatial selection mask for each decomposed large kernel obtained using the sigmoid activation function, *S* represents the attention feature, *F* denotes the convolution layer, and *Y* denotes output feature.

#### GSConv module

In backbone, the spatial downsampling transmits the information to the channel, risking feature loss due to reduced feature map dimensions and increased channel size. The neck, having undergone downsampling, has minimal dimensions but significant channel size, thus containing less redundancy and not needing further compression. Depthwise separable convolution (DSC) is used to disconnect interchannel links while conveying spatial data to accelerate prediction. However, this might lead to semantic loss due to increased channels and reduced spatial dimensions. In contrast, the channel-dense convolution (SC) effectively retains interchannel information. This study introduces GSConv [40], a hybrid of SC and DSC. When applied to the neck of YOLOv8n, GSConv strikes a balance between model lightness and accuracy. It densely computes while preserving interchannel connections, reducing feature loss and time complexity.

**Fig. 5.**
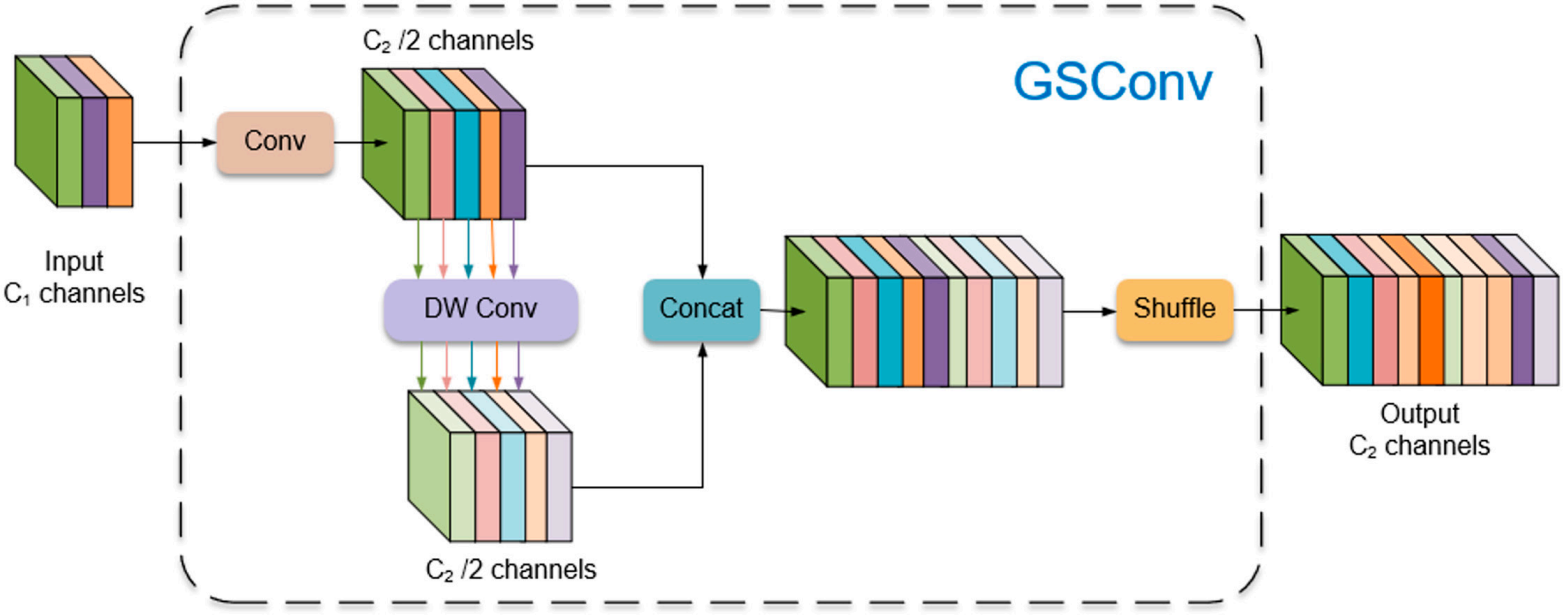
The architecture of the GSConv module.

Figure 5 illustrates the integration of DSC and SC in GSConv through channel shuffling. This method starts by convolving the input feature map with C1 channels to create a C2/2 feature vector. A second C2/2 feature vector is generated through depthwise convolution, and these vectors are then concatenated. Subsequently, channel shuffling is applied to diffuse the information derived from the SC operation across the entirety of information produced by the DSC. This process serves as a uniform mixing technique to seamlessly integrate SC information into the DSC output by evenly exchanging local feature information across different channels.

### Robotic detection and localization systems

**Fig. 6.**
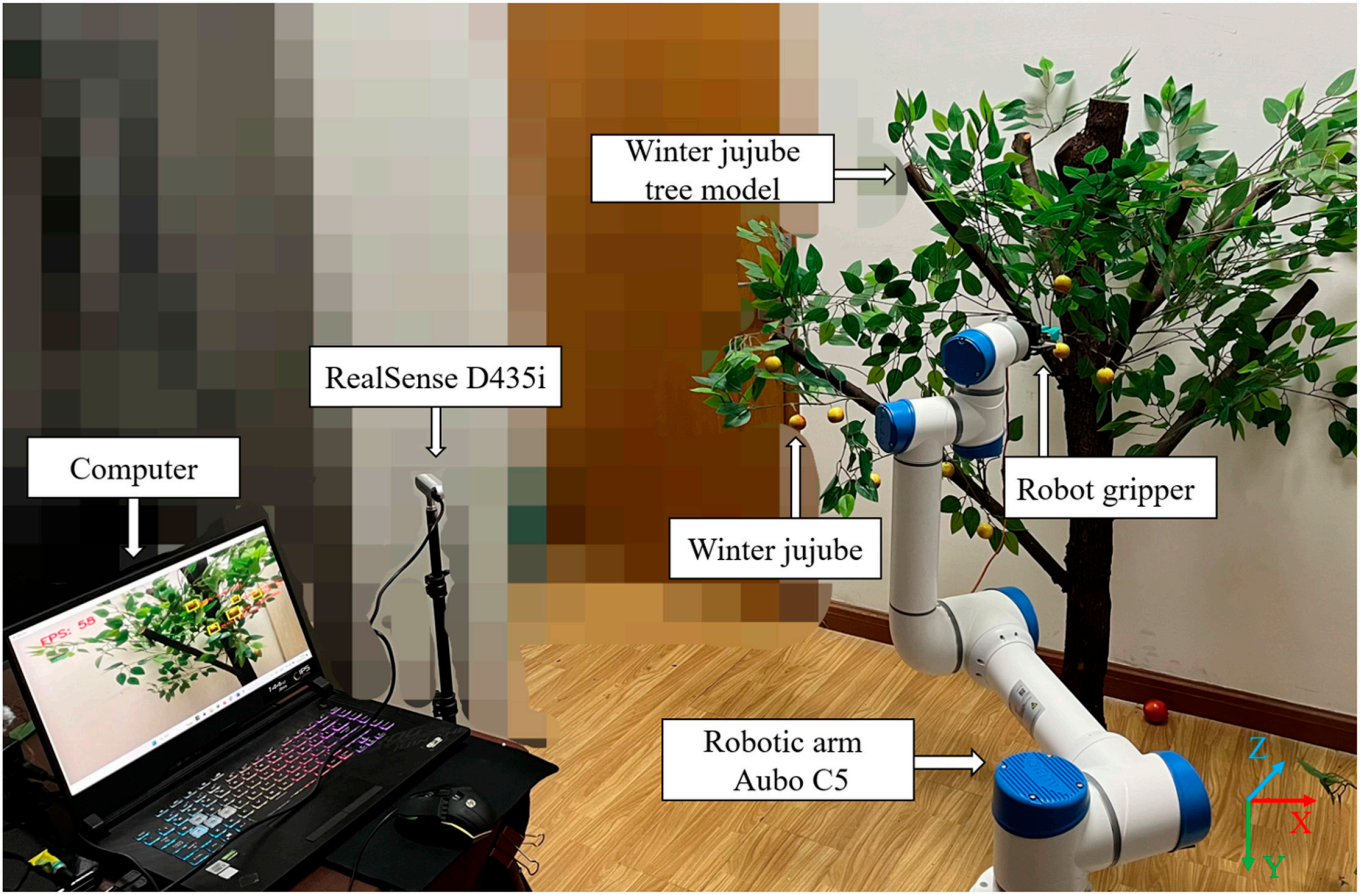
Robotic harvesting system.

**Fig. 7.**
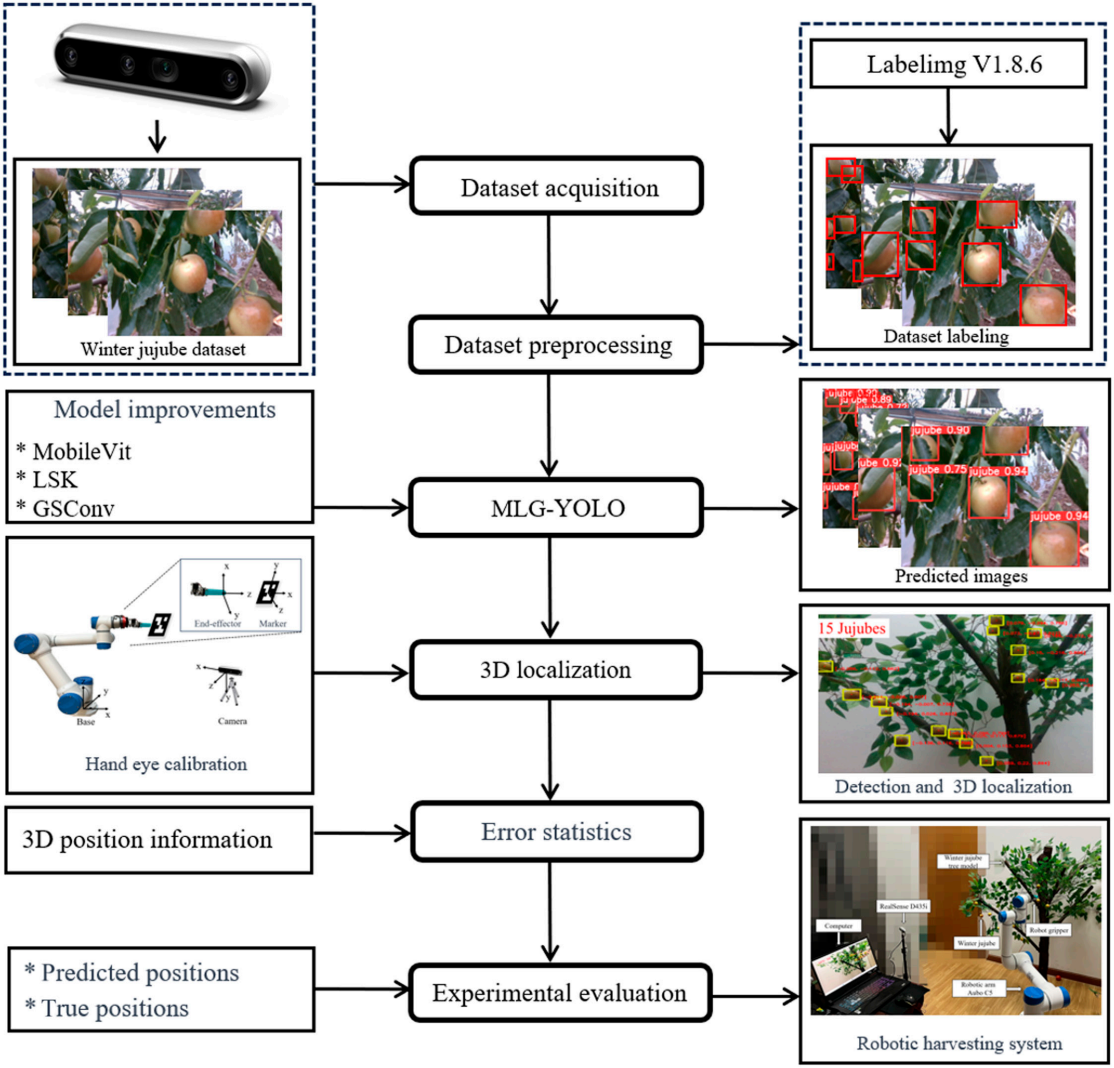
Overall process for detecting and localizing winter jujubes via a robotic harvesting system.

The winter jujube robotic harvesting system developed in this study, depicted in Fig. 6, primarily comprises an AUBO C5 robotic arm (featuring 6 degrees of freedom, a 5-kg load capacity, ±0.1 mm repeat positioning accuracy, and a working radius of 886.5 mm), a RealSense D435i RGB-D camera, a robotic gripper, and a computer. The system’s detection and positioning process, illustrated in Fig. 7, begins with the MLG-YOLO model detecting winter jujubes and obtaining their center point coordinates. The RGB-D camera was then utilized to obtain the depth information of winter jujubes. This information, combined with hand–eye calibration and coordinate transformation, is used to determine their 3D positions. Finally, the robotic arm is controlled to move to the picking position, where the localization accuracy is evaluated. We use a customized robotic gripper to perform the picking action. The gripper system is controlled by an Arduino as the lower unit and a computer as the upper unit, with the Arduino controlling the opening and closing action of the gripper. The gripper is made of flexible material to prevent damage to the fruits during the picking process. The subsequent section details the process of obtaining the 3D positional information of the winter jujubes.

This study deployed the MLG-YOLO model proposed in the “Winter jujube detection based on MLG-YOLO” section within the robotic harvesting system to validate the detection accuracy of the MLG-YOLO algorithm and achieve 3D localization of winter jujubes. After detecting winter jujubes through the model, the center coordinates of the detection frame are used as the positions of the winter jujubes. The formula for calculating the 2D coordinates of the winter jujubes is shown in Eq. 2:

$$C_{o}=\left(\frac{X_{\min}+X_{\max}}{2},\frac{Y_{\min}+Y_{\max}}{2}\right)$$ (2)

where (*X*_min_, *Y*_min_) represents the coordinates of the top-left corner of the detection frame and (*X*_max_, *Y*_max_) represents the coordinates of the bottom-right corner.

Using the Realsense D435i camera, the depth value of the winter jujube’s center point is obtained. The image coordinates are then converted to the camera coordinate system through the Realsense SDK, thus obtaining the coordinates *P*_c_ = (x_c_, y_c_, z_c_) of the winter jujube’s center point in the camera coordinate system. Transforming the camera’s coordinate system to the robot’s base coordinate system is a crucial step in obtaining the 3D localization information of winter jujubes. This coordinate transformation ensures that the robot can accurately locate the position of each winter jujube based on the image data captured by the camera and can effectively perform harvesting in the actual physical space. In this study’s robot vision system, hand–eye calibration and coordinate transformation are performed using ArUco markers and the eye-to-hand configuration method as shown in Fig. S1. The eye-to-hand configuration method provides a broader global view of the harvesting area while not adding a load on the end of the robot arm. During the procedure of hand–eye calibration, the Aruco_ros, Easy_handeye, and Vision_visp function packages are first compiled in ROS on the Linux system. Calibration plate parameters are set using the ArUco marker generator, and a full-size ArUco marker is fixed to the robot’s end effector, allowing it to move with and remain relatively stationary to the end effector. Then, the camera is fixed within the robot’s working area to ensure that it can clearly capture the ArUco markers within the robot’s operating area. By controlling the robot to assume 17 different redundant postures and continuously sampling through the camera, the images of the ArUco markers are captured and the position and orientation of the robot’s end effectors are recorded in the robot’s base coordinate system, and the captured images are processed using the ArUco library to detect the ArUco markers and to compute the relative pose of the ArUco markers with respect to the camera coordinate system. The calibration software eventually calculates the rotation and translation information of the camera’s coordinate system relative to the robot’s base coordinate system, which is represented by a quaternion (*qw*, *qx*, *qy*, *qz*) and a translation vector *t*. The translation vector *t* is shown as follows:

$$t=\left[\begin{array}{c} t_{x}\\ t_{y}\\ t_{z} \end{array}\right]$$ (3)

where *t* describes the displacement of the origin of the camera coordinate system relative to the origin of the robot base coordinate system. *t_x_* represents the displacement in the *x*-axis direction, *t_y_* represents the displacement in the *y*-axis direction, and *t_z_* represents the displacement in the *z*-axis direction.

The rotation matrix *R* corresponding to the quaternion (*qw*, *qx*, *qy*, *qz*) is shown as follows:

$$R=\left[\begin{array}{ccc} 1-2{\mathrm{qy}}^{2}\;-\;2{\mathrm{qz}}^{2} & 2\text{qxqy}\;-\;2\text{qzqw} & 2\text{qxqz}+2\text{qyqw}\\ 2\text{qxqy}\;+\;2\text{qzqw} & 1\;-\;2{\mathrm{qx}}^{2}\;-\;2{\mathrm{qz}}^{2} & 2\text{qyqz}\;-\;2\text{qxqw}\\ 2\text{qxqz}\;-\;2\text{qyqw} & 2\text{qyqz}\;+\;2\text{qxqw} & 1\;-\;2{\mathrm{qx}}^{2}\;-\;2{\mathrm{qy}}^{2} \end{array}\right]$$ (4)

Combining the rotation matrix *R* and the translation vector *t* into a 4 × 4 affine transformation matrix *T*, the affine transformation matrix is shown as follows:

$$T=\left[\begin{array}{cc} R & t\\ 0 & 1 \end{array}\right]$$ (5)

To convert the coordinates of the winter jujube center point in the camera coordinate system *P*_c_ = (x_c_, y_c_, z_c_) to the homogeneous coordinates ${P_{c}}^{\prime }$ = (x_c_, y_c_, z_c_, 1), the affine transformation matrix *T* is used to transform the coordinates and the homogeneous coordinates ${P_{b}}^{\prime }$ are calculated in the robot base coordinate system as shown below:

$${P_{b}}^{\prime }=T\times {P_{c}}^{\prime }$$ (6)

where ${P_{b}}^{\prime }=\left(x_{b},y_{b},z_{b},1\right)$.

By using the above calibration and coordinate transformation methods, the 3D coordinates *P_b_* = (*x_b_*, *y_b_*, *z_b_*) of the winter jujube in the robot base coordinate system are finally obtained, thus achieving 3D localization of the winter jujube.

### Environmental settings

The experimental setup for deep learning in this study includes the use of an NVIDIA GeForce RTX 2060 GPU and an Intel Core i7-10870H CPU, Windows 11 as an operating system, and CUDA 11.6 and CUDNN 8.2.1 for computation acceleration. All the experiments are conducted with 300 epochs of model training under the following hyperparameters: a stochastic gradient descent (SGD) optimizer, a batch size of 4, a learning rate of 0.01, a momentum factor of 0.937, and a weight decay of 0.0005.

For the robotic harvesting system experiments, the setup included Ubuntu 20.04 as the operating system and ROS 1 as the ROS version. The integrated development environment is PyCharm 2023.3.2, and Python version 3.9 is used.

### Evaluation metrics

The performance of object detection algorithms is evaluated via mean average precision (mAP), precision, recall, FLOPs, model size (MB), and parameters. These metrics gauge model accuracy and complexity. Localization error is evaluated in the robot base coordinate system using absolute and standard errors in the *X*, *Y*, and *Z* directions (refer to Fig. 6). Formulas are provided below:

$$m\mathrm{AP}=\frac{1}{C}\sum_{i=1}^{N}P\left(i\right)\;▵\;R\left(i\right)$$ (7)

$$\text{Precision}=\frac{\mathrm{TP}}{\mathrm{TP}+\mathrm{FP}}\times 100\%$$ (8)

$$\text{Recall}=\frac{\mathrm{TP}}{\mathrm{TP}+\mathrm{FN}}\times 100\%$$ (9)

$$\Delta X=\frac{1}{n}\sum_{i=1}^{n}|X_{i}|$$ (10)

$$\Delta Y=\frac{1}{n}\sum_{i=1}^{n}|Y_{i}|$$ (11)

$$\Delta Z=\frac{1}{n}\sum_{i=1}^{n}|Z_{i}|$$ (12)

$$SE=\frac{\sqrt{\frac{\sum_{i=1}^{n}{\left(X_{i}-\bar{X}\right)}^{2}}{n-1}}}{\sqrt{n}}$$ (13)

where *TP* denotes true positive, *FP* denotes false positive, and *FN* denotes false negative. In this study, only one detection category is considered, and the mAP is equivalent to the AP. Δ*X*, Δ*Y*, and Δ*Z* denote the absolute positioning errors in that direction. *X*_i_, *Y*_i_, and *Z*_i_ are the errors of the *i*th measurement point in that direction, $\bar{X}$ is the average error, and n is the number of measurement points.

## Results

### Insertion position of the LSK module

**Fig. 8.**
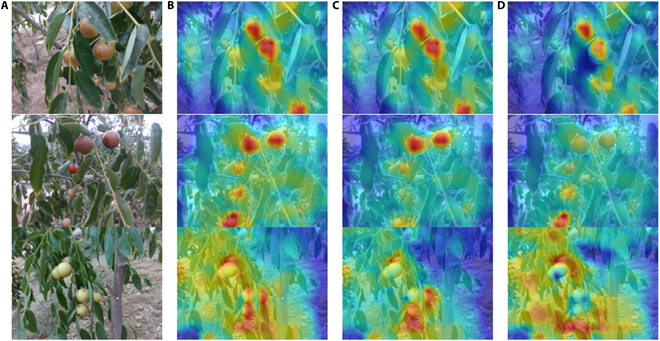
Visual feature mapping of LSKblock at different insertion positions based on CAM technology. (A) Test images. (B) Insertion after the P3 scale processing layer. (C) Insertion after the P4 scale processing layer. (D) Insertion after the P5 scale processing layer.

In this study, the LSK module was inserted into various layers of the neck, specifically following the P3, P4, and P5 scale processing layers. These insertion points, each targeting different scale features with attention mechanism enhancements, aided the model in more effectively extracting features and detecting objects across multiple scales. As illustrated in Table 1, the LSKblock after the P3 scale processing layer in the YOLOv8 network outperformed the P4 and P5 scales in terms of both precision and mAP, with improvements of 2.30%, 3.30%, and 3.10%, respectively, over those of the YOLOv8n model. This particular insertion point, situated after upsampling and concat operations, specifically addressed smaller-scale features. Class activation mapping (CAM) visualizations, as shown in Fig. 8, indicated that the insertion of the LSK block at this location was more effective in focusing on useful feature regions. Based on these findings, this study chose to insert the LSK block after the P3 scale processing layer in constructing the MLG-YOLO architecture and subsequent ablation experiments.

**Table 1.** Quantitative evaluation metrics for different insertion positions of LSKblock. A, B, and C denote the insertion positions of the LSK block. A represents insertion after the P3 scale processing layer, B represents insertion after the P4 scale processing layer, and C represents insertion after the P5 scale processing layer.

YOLOv8n	Insertion points of LSKblock			Precision (%)	Recall (%)	mAP (%)	Parameters	FLOPs (G)
	A	B	C					
√				85.50	80.10	89.20	3,011,043	8.20
√	√			87.80	83.40	92.30	3,022,377	8.30
√		√		85.30	84.10	91.90	3,129,497	8.30
√			√	85.10	84.50	91.90	3,175,605	8.50

### Ablation study

This study used YOLOv8n for the baseline model and incorporating these improvements for performance analysis to evaluate effectiveness of MobileViT backbone, LSKblock, and GSConv in enhancing winter jujube detection. As Table 2 indicates, individually integrating MobileViT, LSKblock, and GSConv each enhanced model performance. Notably, replacing the backbone with MobileViT not only improved performance slightly but also significantly reduced model complexity and size, as evidenced by a 60.77% decrease in parameters, a 2.9-G reduction in FLOPs, and a 57.65% decrease in model size. Inserting the LSKblock in the neck notably enhanced the accuracy and increased the precision, recall, and mAP by 3.30%, 3.30%, and 3.10%, respectively. Adding the GSConv module led to significant improvements in the recall and mAP, with increases of 3.60% and 3.20%, respectively, while slightly reducing the model parameters. The simultaneous integration of both LSKblock and GSConv achieved the highest accuracy, with minimal changes in model size and complexity. Therefore, combining MobileViT effectively enhances the accuracy and weakens the model.

**Table 2.** Ablation experiments with different MLG-YOLO modules

YOLOv8n	Improvement methods			mAP (%)	Recall (%)	Precision (%)	FLOPs (G)	Parameters	Model size (MB)
	MobileVit	LSK	GSConv						
√				89.20	80.10	85.50	8.20	3,011,043	5.95
√	√			91.00	83.90	84.90	5.30	1,181,307	2.52
√		√		92.30	83.40	87.80	8.30	3,022,377	5.98
√			√	92.40	83.70	86.60	8.10	2,921,283	5.79
√	√	√		92.50	85.00	85.70	5.40	1,185,505	2.53
√	√		√	91.80	84.80	85.20	5.30	1,170,511	2.51
√		√	√	92.30	82.60	87.90	8.20	2,932,617	5.82
√	√	√	√	92.70	84.50	86.80	5.40	1,173,429	2.52

**Fig. 9.**
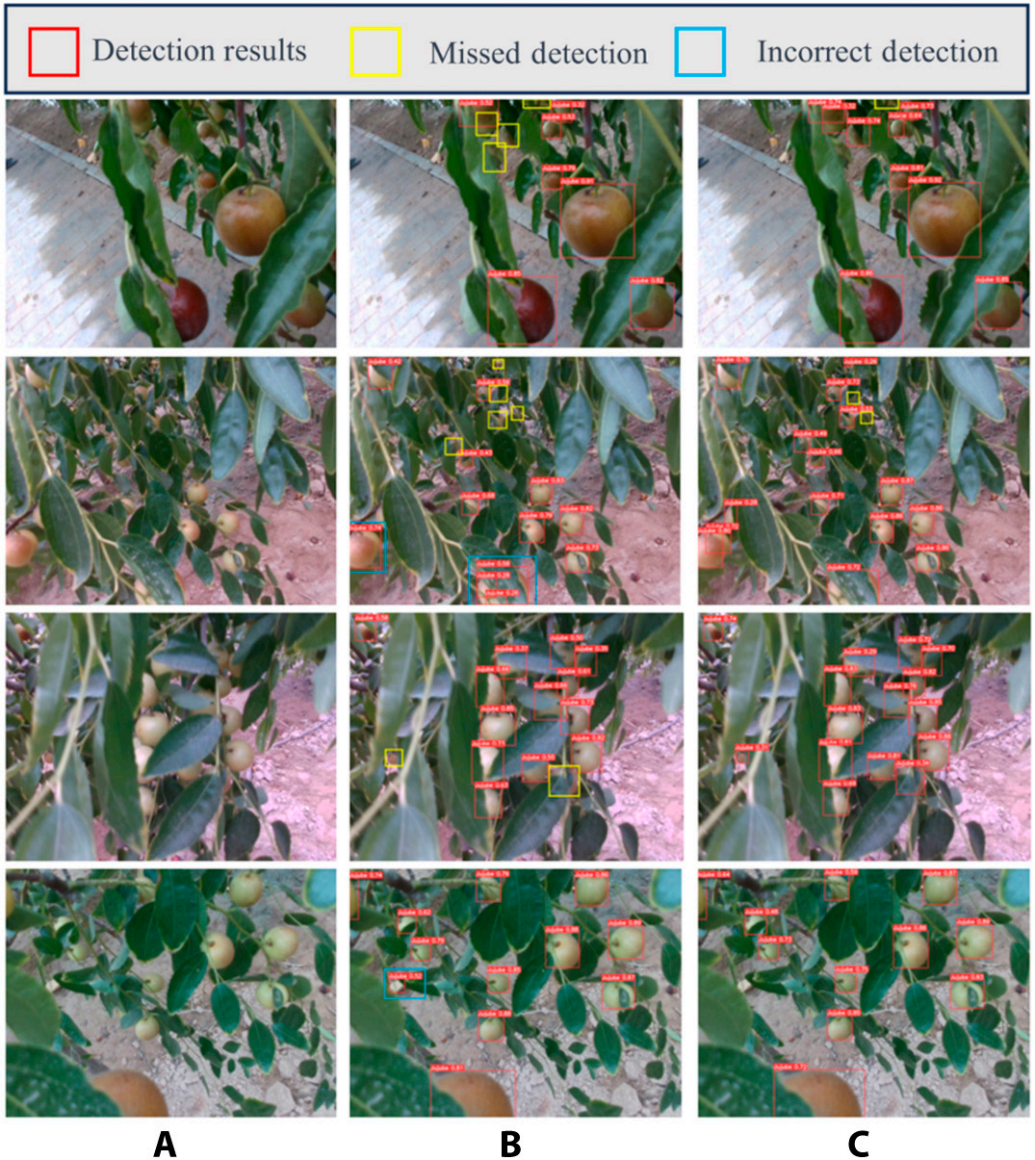
Comparison of the detection effect of model improvement. (A) Test image. (B) YOLOv8n. (C) MLG-YOLO.

We compared the detection outcomes before and after model improvements under various challenging scenarios. The model’s detection results are indicated by red boxes, with missed detections in yellow and false detections in blue, as shown in Fig. 9. The YOLOv8n model exhibited numerous missed detections under low-light conditions and when jujubes were obscured by leaves, likely due to difficulty in feature extraction. For overlapping or leaf-obscured winter jujubes, the YOLOv8n model had many missed and false detections, possibly due to inadequate resolution for dense and small objects and obscured key feature information. In contrast, the MLG-YOLO model performed better in these complex scenarios because the LSKblock and GSConv modules, which enhanced feature extraction and context information utilization, effectively captured local features and focused on key object areas, thus enabling more flexible detection in complex situations such as occlusions and overlaps.

### Comparative experiments

To thoroughly compare the overall performance of the MLG-YOLO model and validate its effectiveness in detecting winter jujubes, this study conducted a comparative analysis with other models, including RT-DETR-L [41], SSD, Faster R-CNN, YOLOv4-tiny [42], YOLOv5n, YOLOv7-tiny, and YOLOv8n.

Quantitative evaluation: Table 3 shows the evaluation metrics of various object detection models. In precision, MLG-YOLO outperforms the other models in terms of both the recall and mAP, achieving accuracies of 84.50% and 92.70%, respectively. Among the competing models, YOLOv7-tiny has the highest recall of 82.29%, while YOLOv5n has the highest mAP of 89.20%. In terms of precision, MLG-YOLO also outperformed YOLOv7-tiny by 86.80% and 87.49%, respectively. Regarding model complexity, MLG-YOLO has the fewest parameters (1,173,429), which is significantly lower than those of the other models, and its FLOPs are 5.40 G, just behind YOLOv5n’s 4.50 G. Additionally, the size of the MLG-YOLO model is the smallest among all models, at only 2.52 MB. In summary, MLG-YOLO demonstrates the highest accuracy in detecting winter jujubes within intricate orchard settings while also maintaining fewer parameters, minimal model size, and low FLOPs, striking an optimal balance between detection accuracy and speed.

**Table 3.** Performance comparison between mainstream object detection models

Models	mAP (%)	Recall (%)	Precision (%)	FLOPs (G)	Parameters	Model size (MB)
Faster R-CNN	68.41	60.34	72.56	948.10	137,098,724	108.17
SSD	75.76	62.50	81.40	87.40	26,285,486	90.60
RT-DETR-L	87.50	78.00	82.60	110.00	32,808,131	63.00
YOLOv4-tiny	85.76	80.24	83.38	31.70	6,056,606	23.57
YOLOv5n	89.20	81.60	86.30	4.50	1,760,518	3.74
YOLOv7-tiny	88.42	82.29	87.49	13.20	6,007,596	12.29
YOLOv8n	89.20	80.10	85.50	8.20	3,011,043	5.95
MLG-YOLO	92.70	84.50	86.80	5.40	1,173,429	2.52

**Fig. 10.**
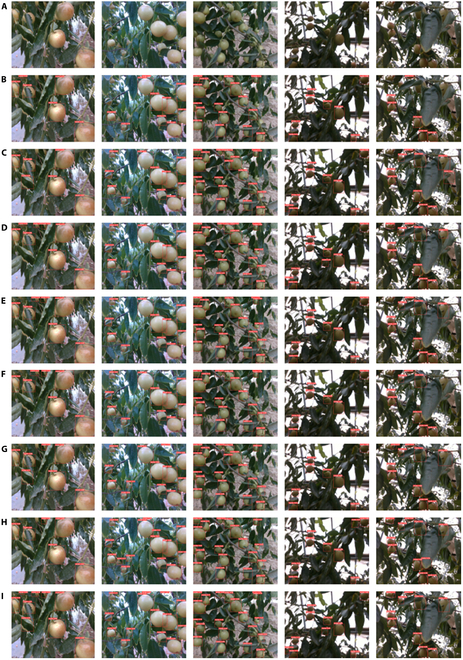
Effect of mainstream object detection models on detecting winter jujubes. (A) Test images. (B) Faster R-CNN. (C) SSD. (D) RT-DETR-L. (E) YOLOv4-tiny. (F) YOLOv5n. (G) YOLOv7-tiny. (H) YOLOv8n. (I) MLG-YOLO.

Qualitative evaluation: For a more nuanced assessment, we selected typical images of winter jujubes in natural environments to test the performances of different models under various conditions, such as light changes, leaf shading, and winter jujubes. As illustrated in Fig. 10, all models exhibit some degree of detection leakage for winter jujubes obscured by leaves or smaller in size due to less obvious features. Among them, MLG-YOLO shows the least detection leakage. There are instances where winter jujubes covered by leaves are detected, but the detection frame does not entirely and accurately encompass the full contour of the winter jujubes, impacting the localization precision. In low-light images, where the winter jujubes blend with the background or are partially obscured, several models, such as SSD and YOLOv8n, incorrectly detect the background as winter jujubes. The others missed detections altogether. Overall, while all the models can detect winter jujubes with distinct features, MLG-YOLO outperforms the other models in identifying less obvious winter jujubes.

The combined quantitative and qualitative evaluations confirm that our proposed MLG-YOLO model outperforms other mainstream object detection models in terms of performance. By analyzing the performance metrics in Table 3 and examining the detection results in Fig. 10, we conclude that the improvement strategy in this study effectively enhances the model’s detection accuracy and achieves lightweighting. MLG-YOLO, with its backbone reconfigured using MobileViT, is more efficient than other lightweight models, such as YOLOv4-tiny, YOLOv5n, and YOLOv7-tiny. Additionally, the integration of LSKblock and GSConv bolsters the model’s ability to detect winter jujubes with inconspicuous features, significantly reducing missed and incorrect winter detection.

### Evaluation of 3D localization errors in winter jujubes

This study selected a winter jujube tree model in a laboratory setting as the experimental subject to validate the efficacy of the winter jujube detection and localization methods outlined in the “Robotic detection and localization systems” section, which combines the MLG-YOLO model with an RGB-D camera. The robotic harvesting system, as shown in Fig. 6, was employed for the winter jujube localization experiment to assess the positioning accuracy of the robotic arm in a practical operational environment. The detection and localization results of the winter jujubes are shown in Fig. S2, where the detection success rate of 10,15,20,25 winter jujubes in the laboratory environment reached 100%, and the model detection was excellent, which is consistent with the detection results of winter jujubes in the structured orchard environment. The specific experimental procedure was as follows: First, based on the method described in Section 2.3, the RGB-D camera was used to get the position data of the winter jujube center point, which was then set as the measurement point. Subsequently, the robotic arm’s end effector was controlled to move to the predetermined measurement point, and a ruler was used to measure the actual distance between the end effector and the preset point. The measurement data for each point were recorded. The actual measured values were compared with the target position values set in the robotic arm’s control system, and the corresponding errors were calculated. Each measurement point was subjected to 5 repeated experiments, and the average error was calculated to ensure data reliability. A total of 10 sets of random jujube positions were selected for testing. The experimental results showed that the proposed localization method had average positioning errors of 4.20, 4.70, and 3.90 mm in the *X*, *Y*, and *Z* directions, respectively, with standard errors of 1.55, 1.62, and 1.39 mm in these directions, meeting the requirements for the precise localization of winter jujubes. To further validate the accuracy of the method for winter jujube object detection and localization, harvesting tests were conducted in a laboratory environment using a robotic harvesting system. The harvesting process is depicted in Fig. S3. The method can achieve real-time detection and localization of winter jujubes and provides satisfactory picking points in most cases.

## Discussion

We compared the MLG-YOLO model with the results of other recent jujube detection studies. The main methods for jujube detection in recent years have been single-stage object detection algorithms, which focus on improving jujube detection precision and reducing the size or complexity of the model. Liu et al. proposed the YOLOv3-SE model for winter jujube detection, with a mAP of 82.01%, a recall of 83.80%, and a precision of 88.71%. Feng et al. introduced an improved YOLOv5s model for detecting winter jujubes, with a mAP of 90.80%, a recall of 82.00%, and a precision of 88.70%. In comparison, our proposed MLG-YOLO model achieved a recall of 84.50% and a mAP of 92.70%, showing superior detection performance. Li et al. developed an improved SSD model for detecting Lingwu long jujube. The model’s parameter count was 3.62 × 10^6^, while our model’s parameter count was only 1.17 × 10^6^, just 32.32% of theirs, significantly reducing model complexity while maintaining detection accuracy. Our model outperforms other similar jujube detection methods in terms of detection accuracy and lightweight design. To further validate the detection performance of our proposed MLG-YOLO model in complex orchard environments, we detected winter jujubes under different conditions, such as leaf shading and varying light levels, on typical full-size jujube trees in a structured orchard, as shown in Fig. S4. We detected 16 jujube trees, and the fitted regression model, shown in Fig. S5, achieved an average detection accuracy of 92.82% with an *R^2^* of 0.97, demonstrating the effectiveness of our approach in complex orchard environments.

The robot localization process is a critical component in achieving precise detection and localization of small-object fruit. Our proposed method employed the MLG-YOLO model to detect winter jujubes using an RGB-D camera, which captured the center point of each winter jujube as the measurement point for localization experiments. The robotic arm’s end-effector was then controlled to move to these predetermined measurement points. To assess the accuracy of this movement, we measured the actual distance between the end-effector and the target point using a ruler. Multiple experiments were conducted to minimize errors. The results show that the average positioning error of jujube in the *X*, *Y*, and *Z* directions is less than 5 mm. In related research on fruit localization, Liu et al. [43] proposed an apple localization method combining YOLO-V5 and LiDAR with an average localization error of 21.1 mm. Compared to fruit localization methods based on LiDAR, our study’s approach using an RGB-D camera is more cost-effective and achieves greater accuracy. Among methods that also use RGB-D cameras for fruit localization, Ning et al. [44] proposed an improved YOLOv4 and RGB-D camera-based method for detecting and locating bell peppers, with an average localization accuracy of 89.55%. Hu et al. developed an apple localization method using an improved YOLOX and RGB-D camera, with localization errors less than 7 mm in all 3 directions, respectively. Compared to fruits such as apples and bell peppers, winter jujubes are smaller in size, making detection and localization more challenging, and our proposed method shows excellent performance in the localization experiments.

Our proposed method still has some limitations. As shown in Fig. S6, in certain scenarios, the method may fail to correctly detect winter jujubes (as in Fig. S6A) or erroneously identify leaves similar in shape to winter jujubes as the fruit (as in Fig. S6B). This issue can be attributed to reduced image quality due to poor lighting, where key features of the winter jujubes, like texture and color, are not sufficiently distinct, making it challenging for the model to differentiate between winter jujubes and other similarly shaped objects, especially when their sizes are comparable. To address this issue, our future work will consider incorporating more winter jujube images taken under low-light conditions, and images where the texture or color of the winter jujubes closely resembles that of the background to refine the model.

## Conclusion

This study explored an accurate and real-time method for detecting and locating winter jujubes in a complex natural orchard settings. Using the combined MLG-YOLO model and RGB-D camera method proposed in this study, we successfully achieved accurate detection and localization of winter jujube, and the specific conclusions of the research are as described below:

1. A lightweight MLG-YOLO model for detecting winter jujubes was proposed. By reconstructing the backbone of YOLOv8n using MobileViT, the model’s parameters and FLOPs were notably reduced, and the model size was only 2.52 MB, which made the model lightweight and met the requirements for deployment in robotic harvesting systems.

2. To enhance the detection accuracy of the model, the LSKblock and GSConv modules were introduced in neck. The ablation study results showed that compared with those of the YOLOv8n model, the precision, recall, and mAP of the improved model increased by 1.30%, 4.40%, and 3.50%, respectively. In addition, a comparison of the experimental results with those of other mainstream object detection models demonstrated that the enhanced model substantially decreased the number of missed detections of winter jujubes and was more effective at detecting winter jujubes in complex environments.

3. A 3D positioning method for winter jujubes was proposed by combining MLG-YOLO and an RGB-D camera. The results of the 3D positioning error evaluation indicated the localization error of this method was less than 5 mm in all 3 directions, indicating the precise positioning of the winter jujubes. This study offers effective technical assistance for detection and localization of winter jujubes in robotic harvesting within complex environments and can provide a reference for other jujubes or small-object fruits.

## Data Availability

The YOLOv8 model and its derivatives, all model weights and code, including source code and pipelines for hardware that may be involved, are subject to the AGPLv3 license. When sharing the data, model weights, and code used in the research in this paper, we will ensure that we comply with the requirements of the AGPLv3 license and encourage other researchers to comply with the appropriate licenses when using these resources as well. We recognize the importance of open data and code in advancing scientific research. The dataset used in this study has been uploaded to GitHub at https://github.com/SomnuuusY/dataset-jujubes.git.
