# Supplementary material for: MLG-YOLO: A Model for Real-Time Accurate Detection and Localization of Winter Jujube in Complex Structured Orchard Environments
Source: Plant Phenomics. 2024 Sep 23;6:0258. doi: 10.34133/plantphenomics.0258 (PMC11418275; doi:10.34133/plantphenomics.0258)
Supplement: Supplementary 1 — Figs. S1 to S6 [file plantphenomics.0258.f1.zip › Supplementary Materials.docx]

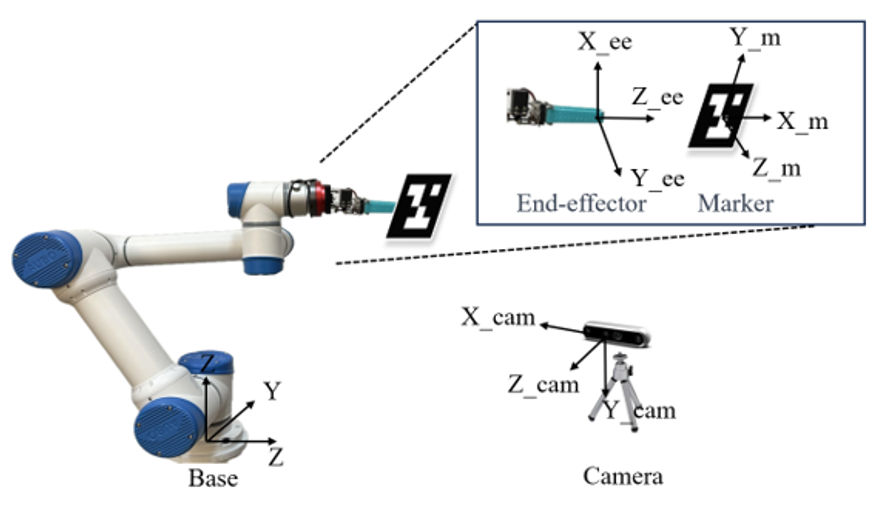


**Figure S1.** Coordinate system relationships of the eye-to-hand calibration method.


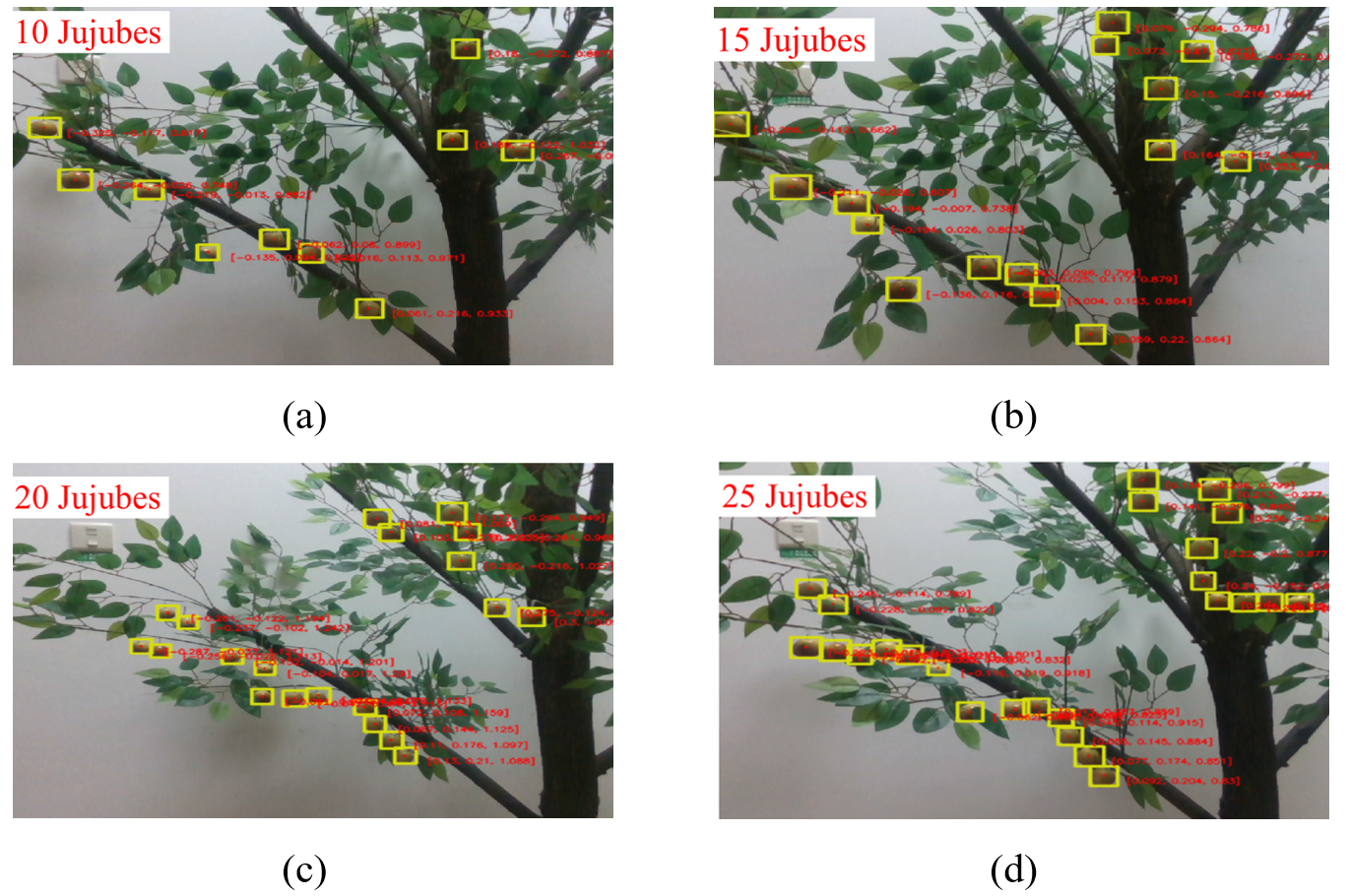


**Figure S2.** Localization results for winter jujubes: (a) 10 winter jujubes, (b) 15 winter jujubes, (c) 20 winter jujubes, and (d) 25 winter jujubes.


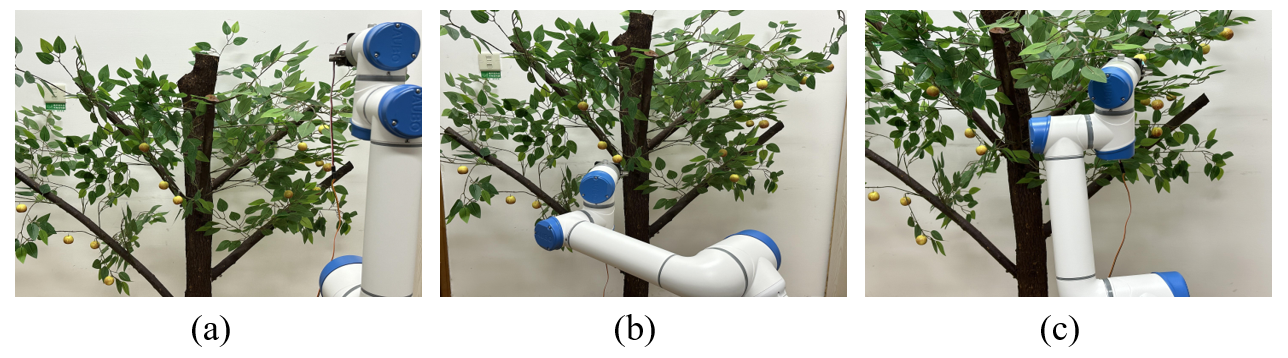


**Figure S3.** Winter jujube harvesting process. (a) Initial position, (b) arrival at the winter jujube picking position, and (c) arrival at the next picking position.


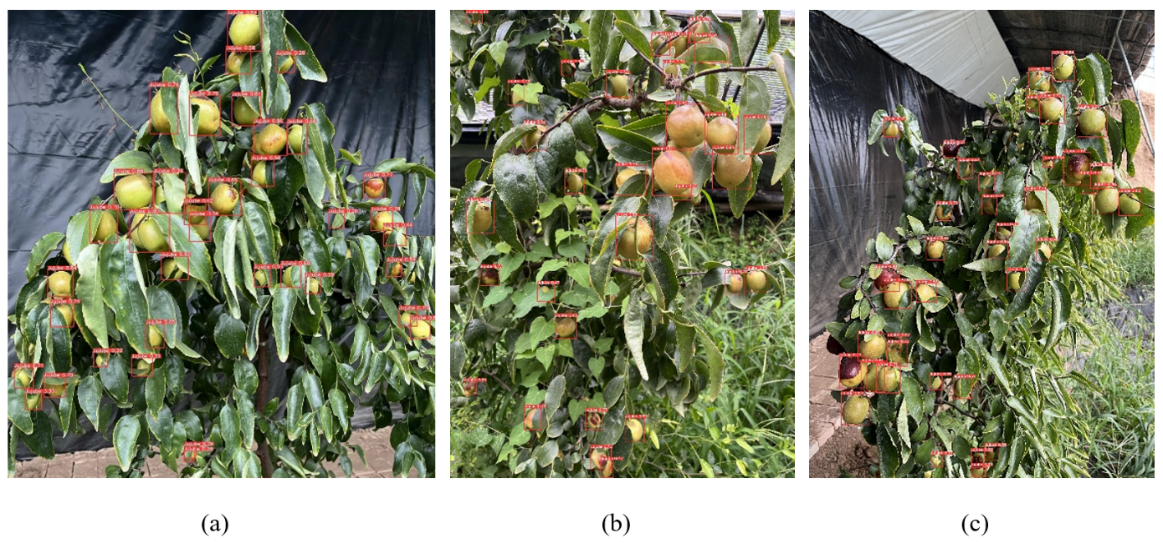


**Figure S4.** Detection effects of typical full-scale winter jujube trees samples. (a) Winter jujubes obscured scene, (b) Scene with weeds in the background, (c) Scenes with low light.


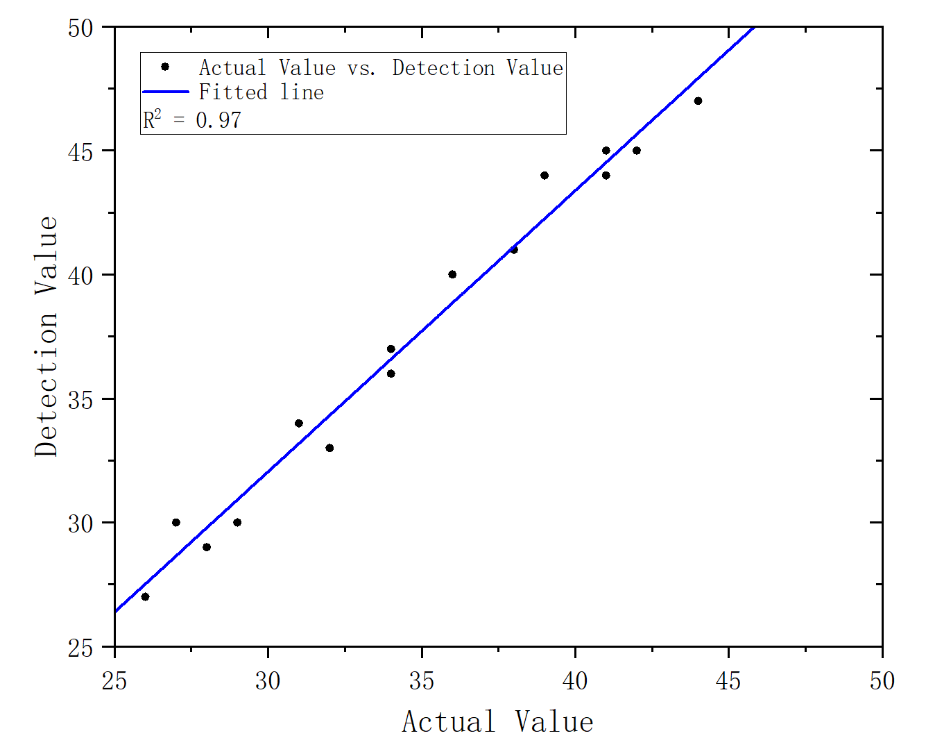


**Figure S5.** Regression modeling of actual value and detection value.


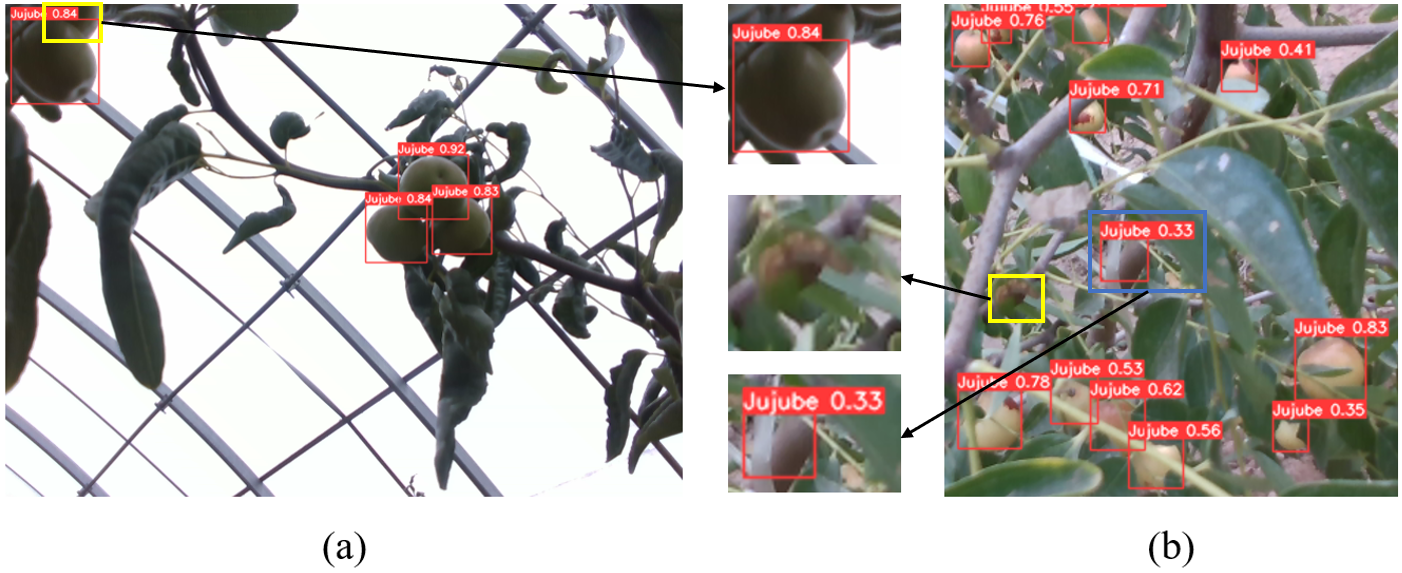


**Figure S6.** False detection due to insufficient light, (a) missed detection case, (b) incorrect detection case.
