# Supplementary figures and images for: MLG-YOLO: A Model for Real-Time Accurate Detection and Localization of Winter Jujube in Complex Structured Orchard Environments
Source: Plant Phenomics. 2024 Sep 23;6:0258. doi: 10.34133/plantphenomics.0258 (PMC11418275; doi:10.34133/plantphenomics.0258)

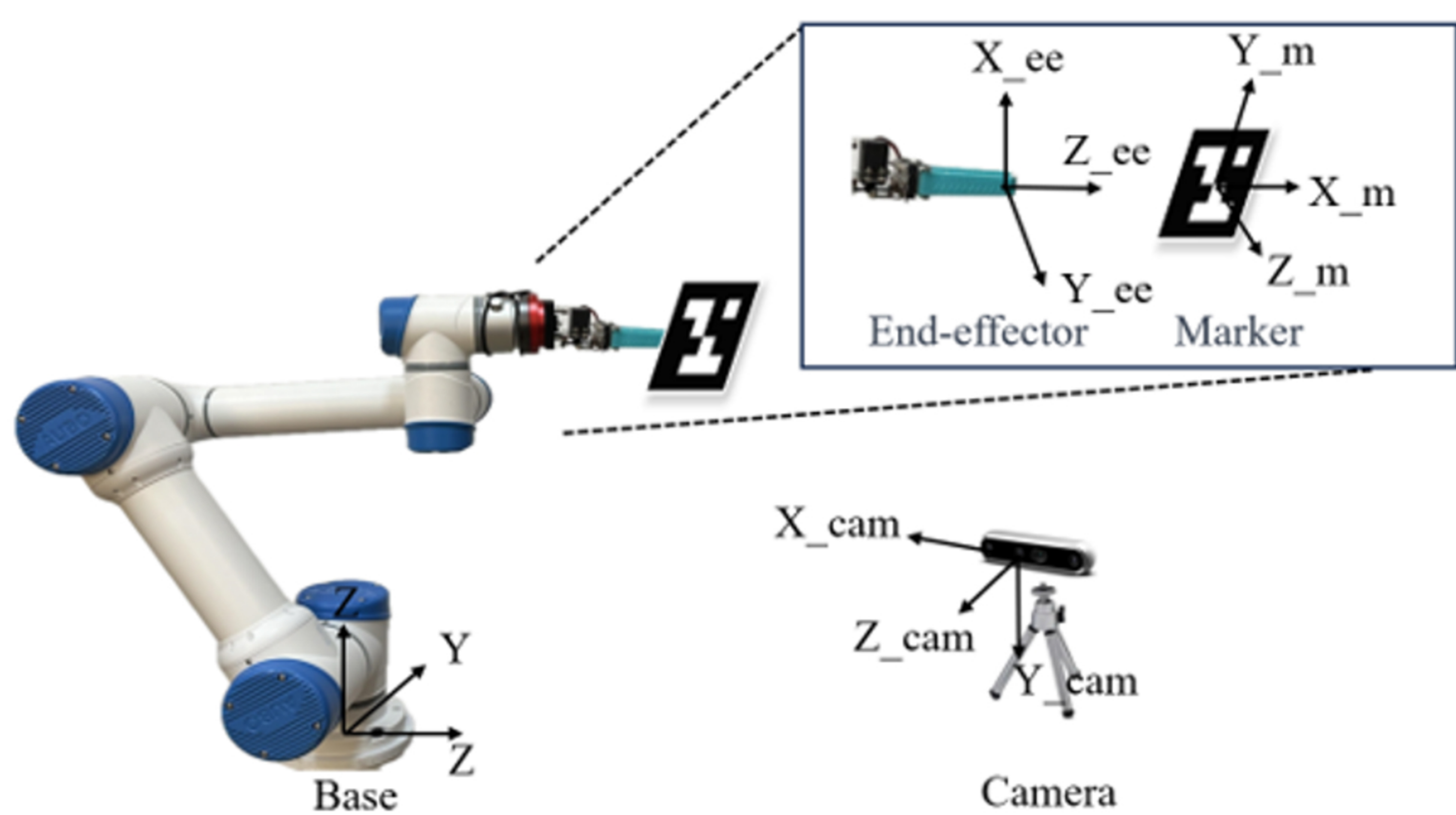

Supplement: Supplementary 1 — Figs. S1 to S6 [file plantphenomics.0258.f1.zip › figure S1.pdf]

10 Jujubes

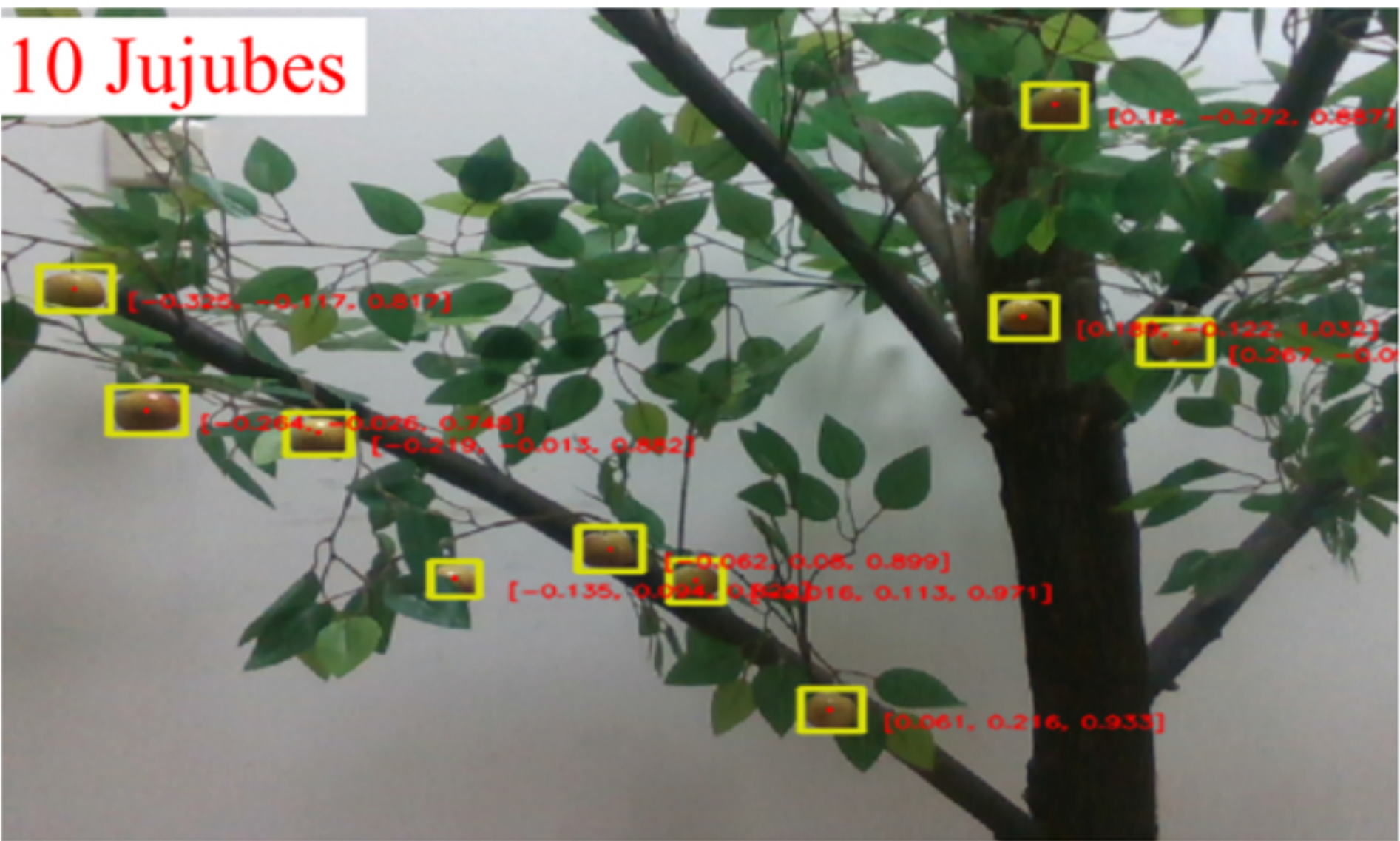

(a)

15 Jujubes

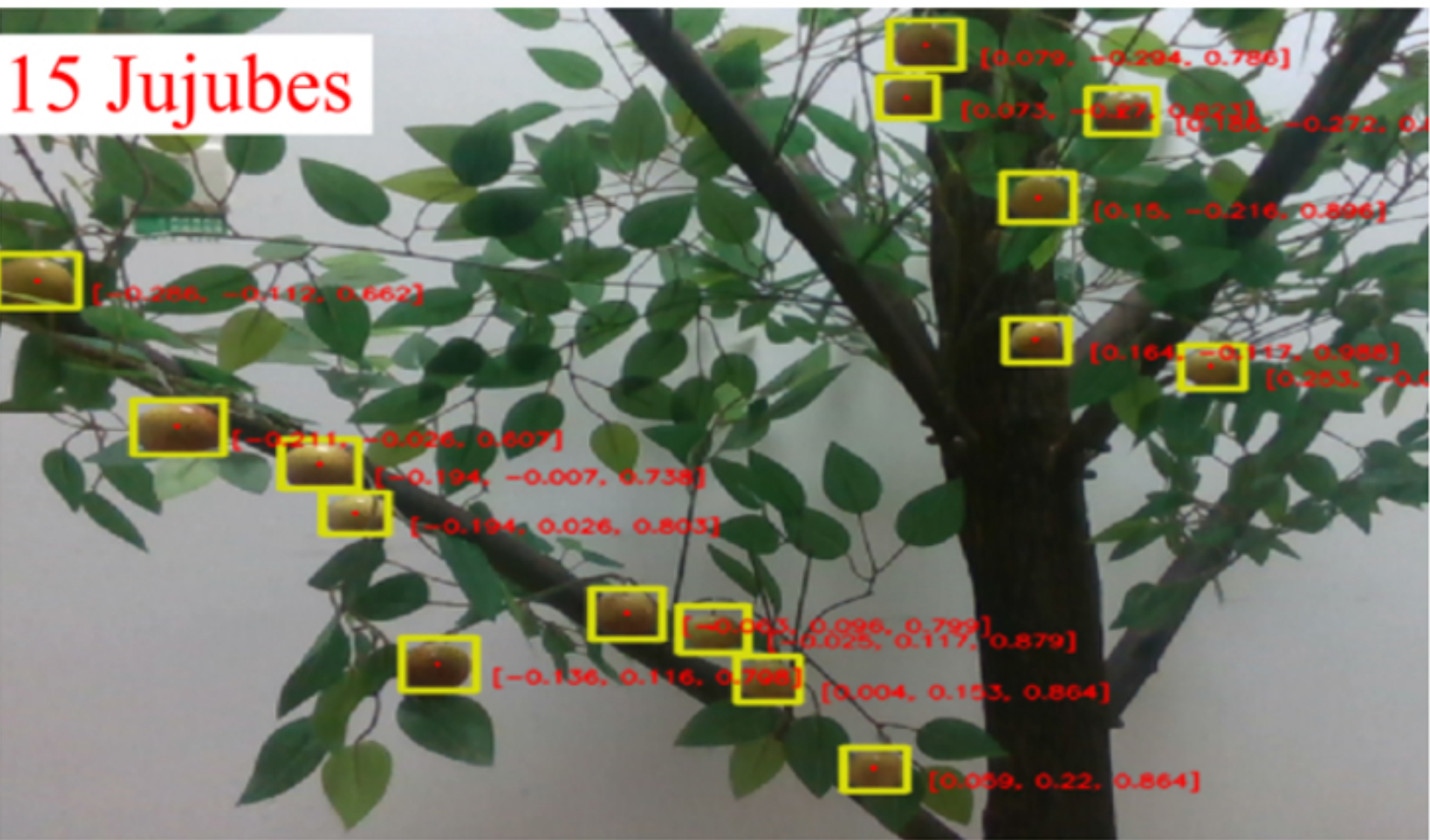

(b)

20 Jujubes

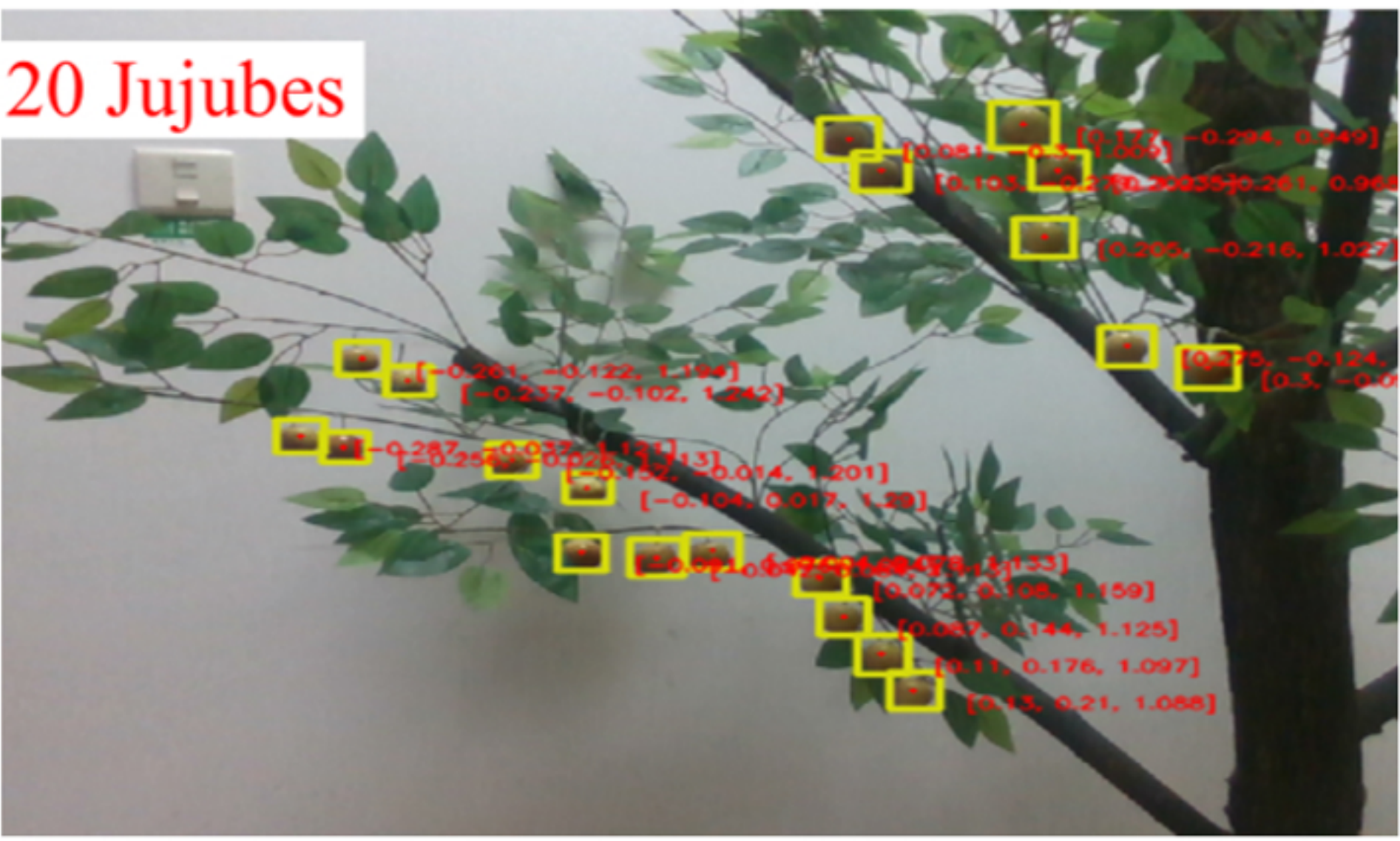

(c)

25 Jujubes

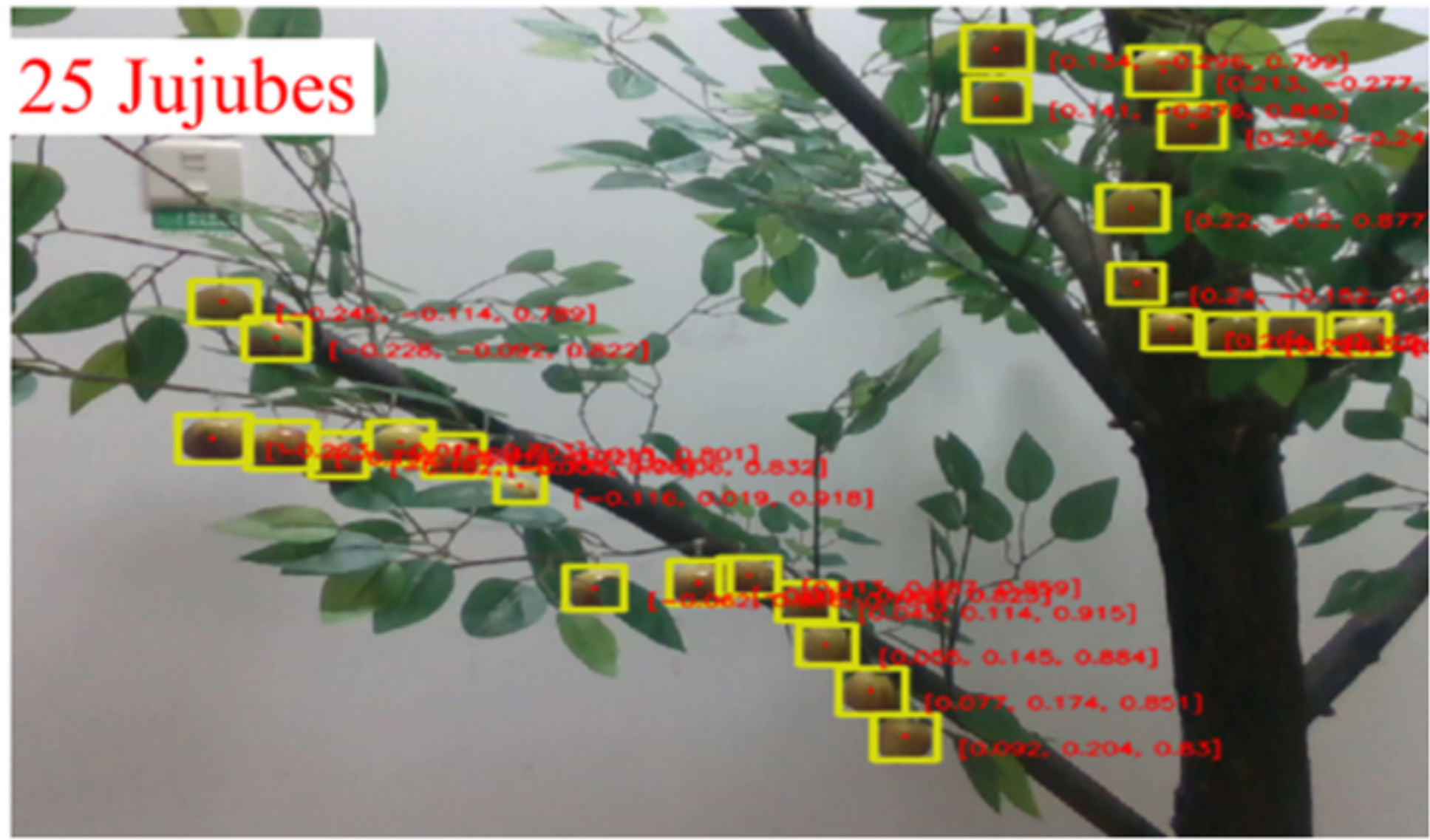

(d)

Supplement: Supplementary 1 — Figs. S1 to S6 [file plantphenomics.0258.f1.zip › figure S2.pdf]

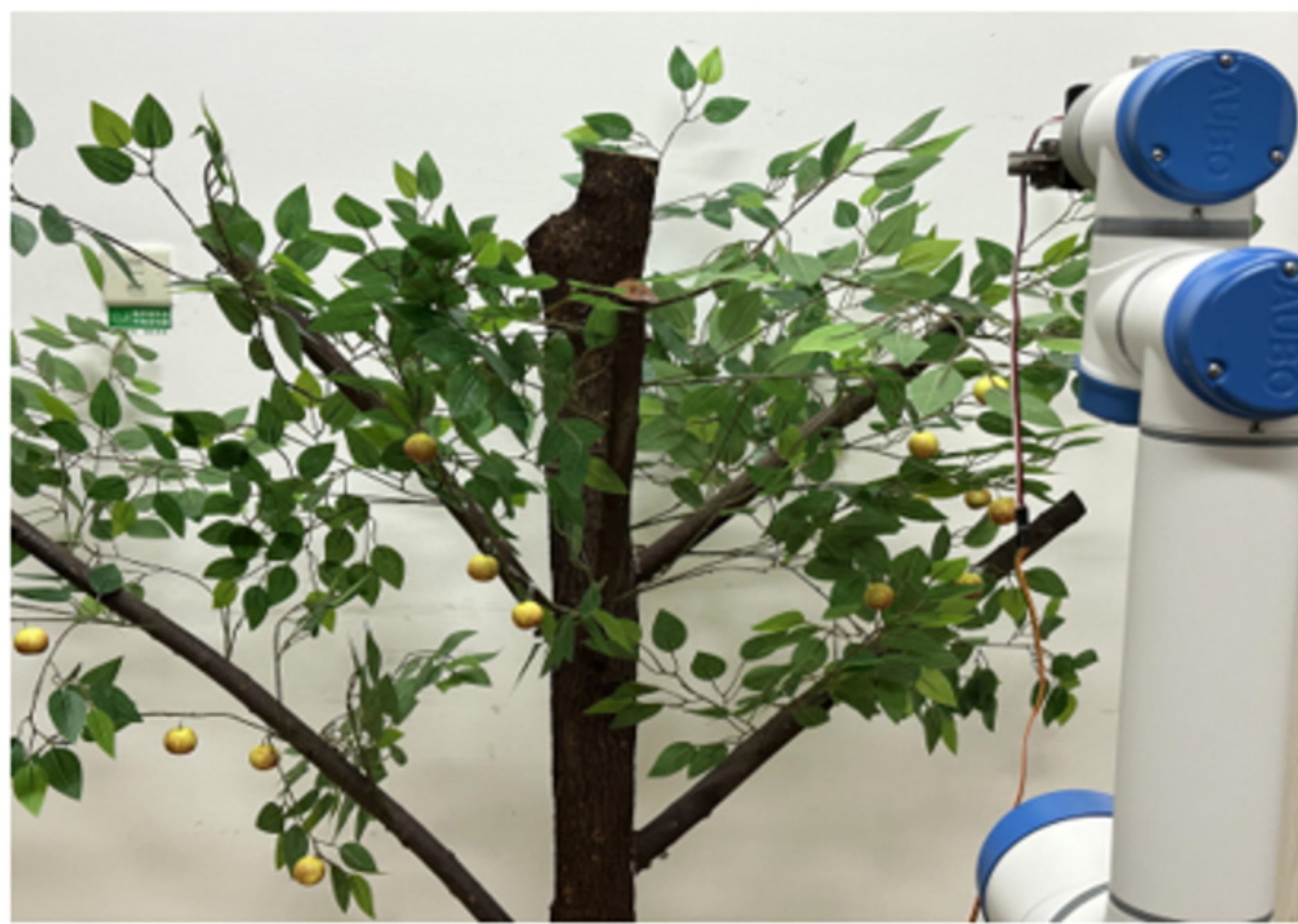

(a)

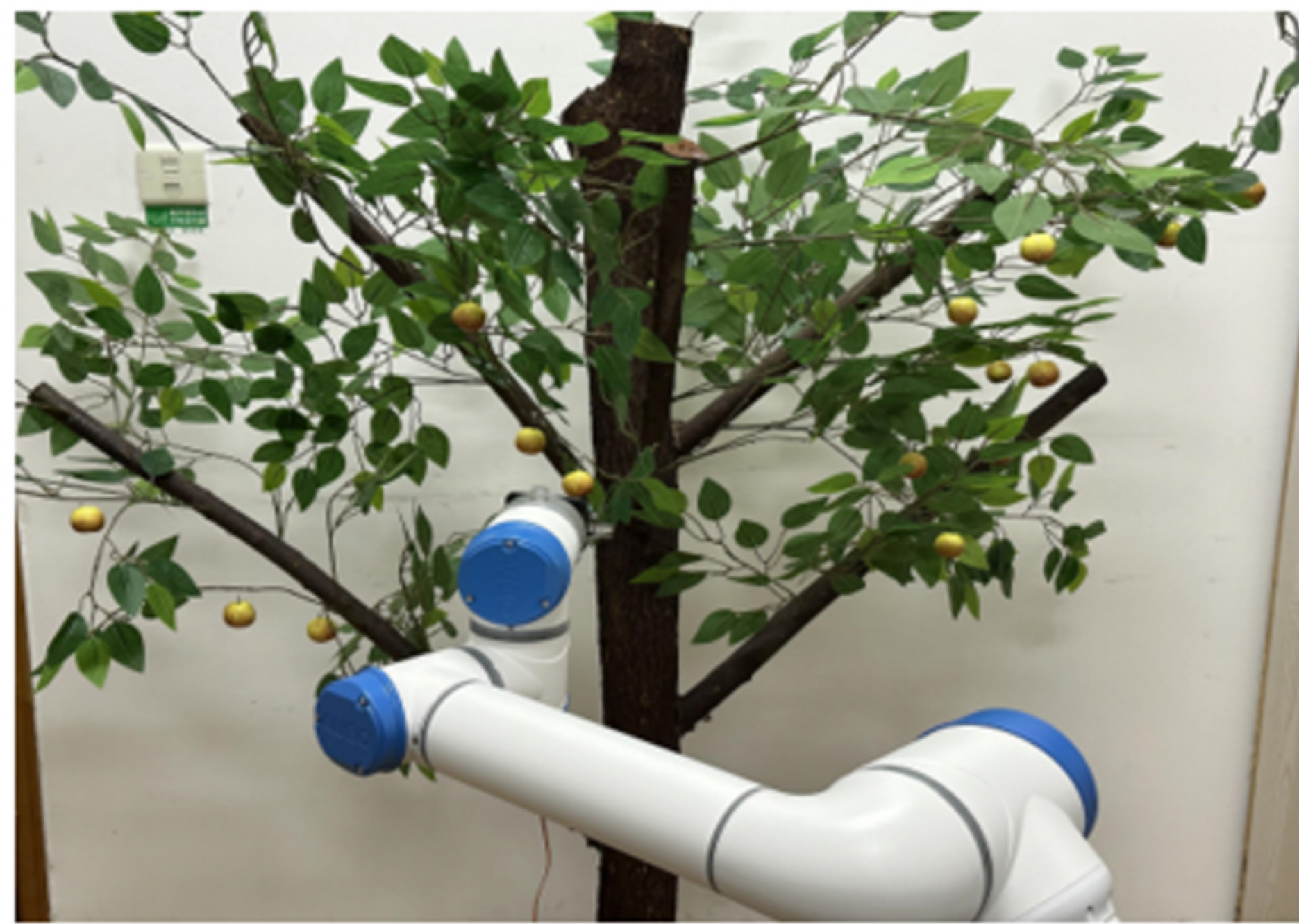

(b)

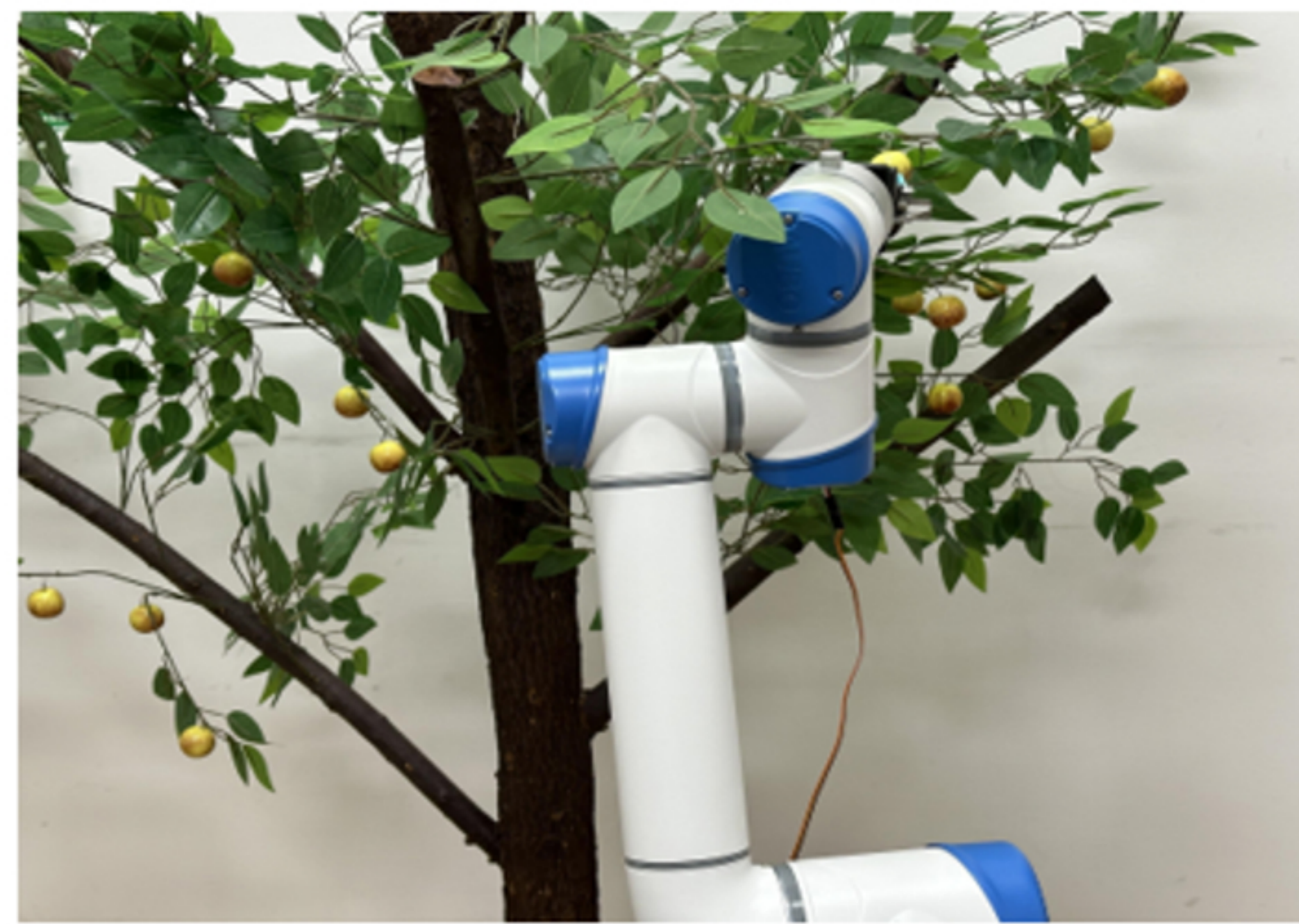

(c)

Supplement: Supplementary 1 — Figs. S1 to S6 [file plantphenomics.0258.f1.zip › figure S3.pdf]

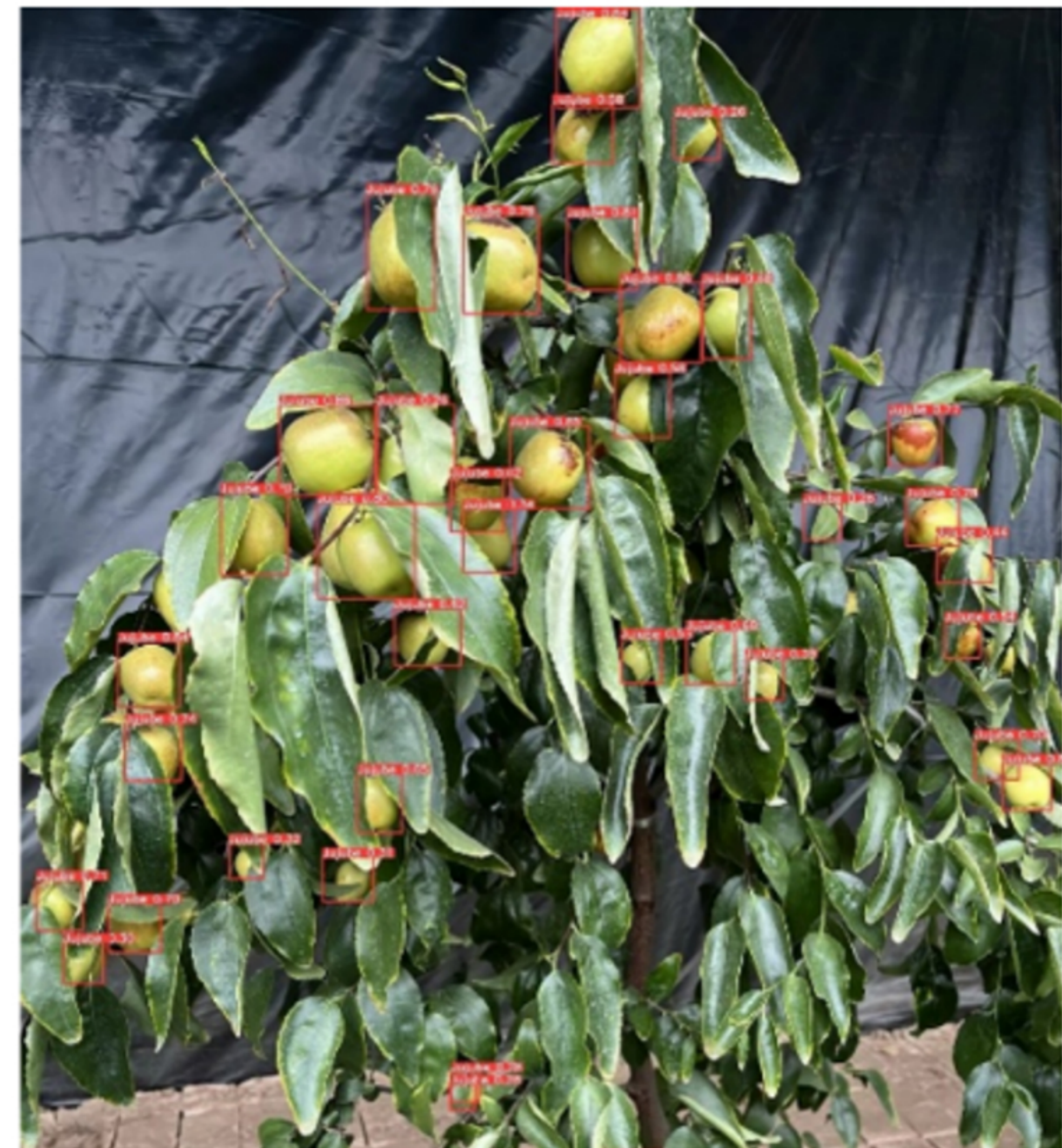

(a)

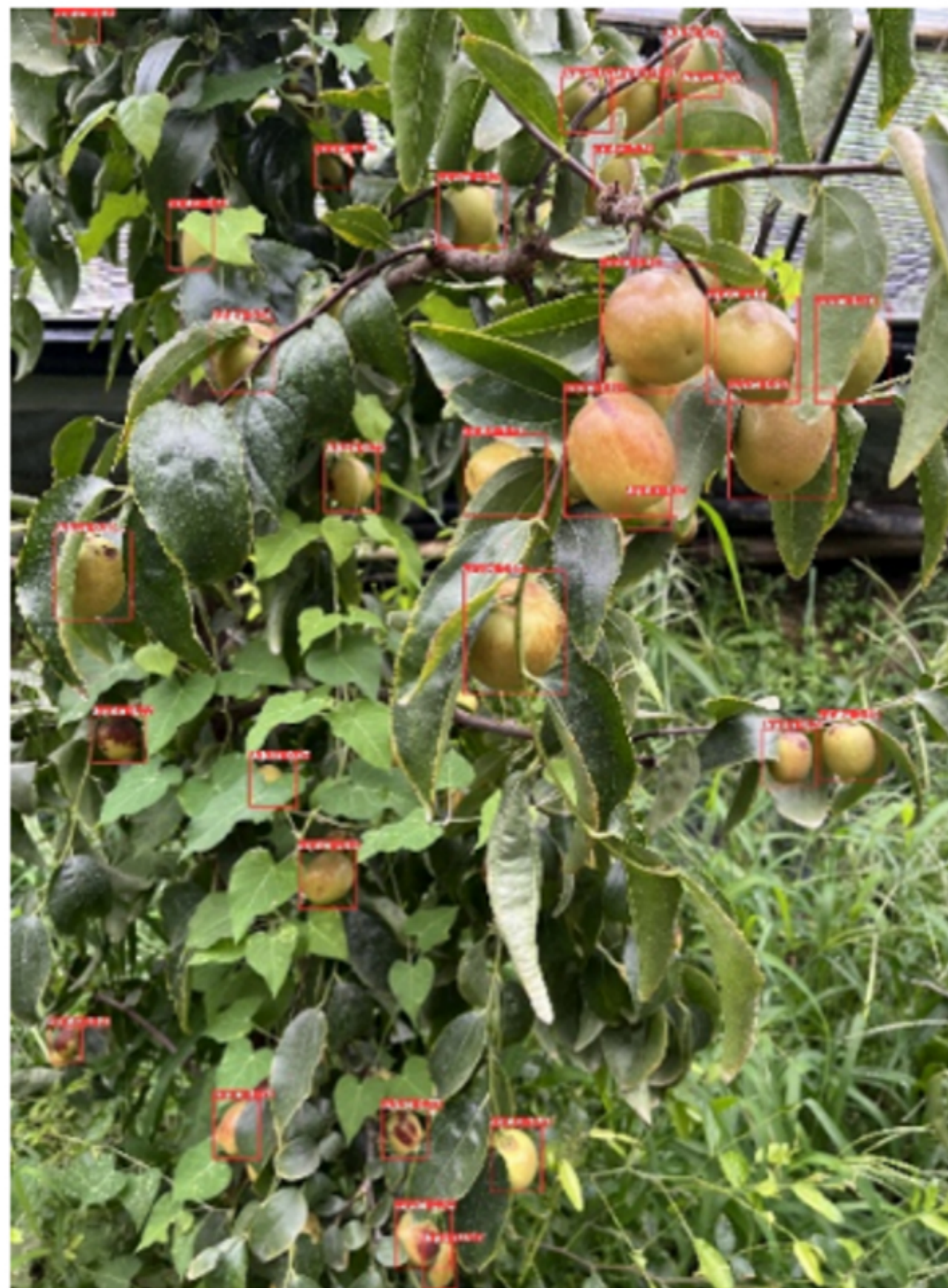

(b)

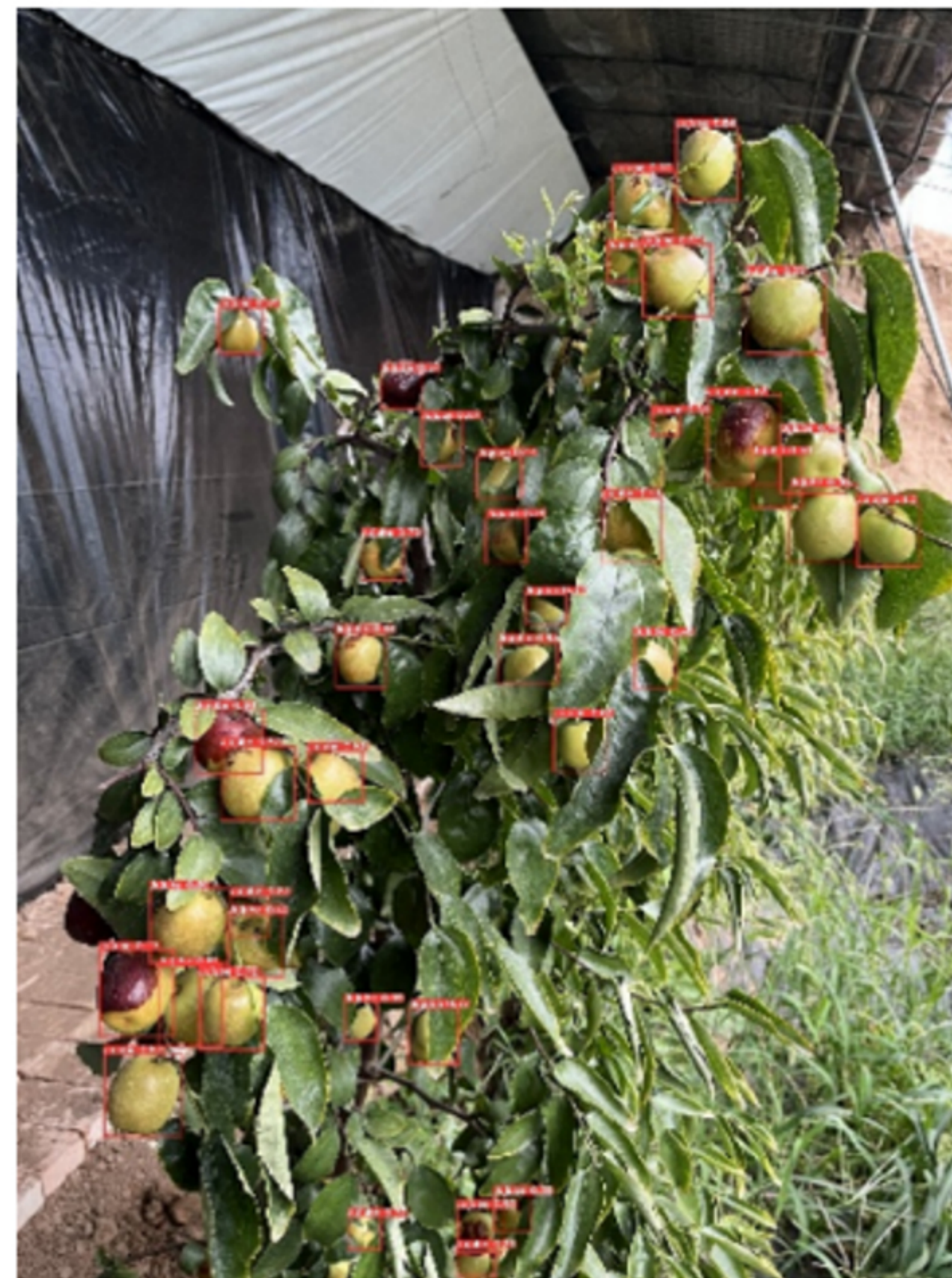

(c)

Supplement: Supplementary 1 — Figs. S1 to S6 [file plantphenomics.0258.f1.zip › figure S4.pdf]

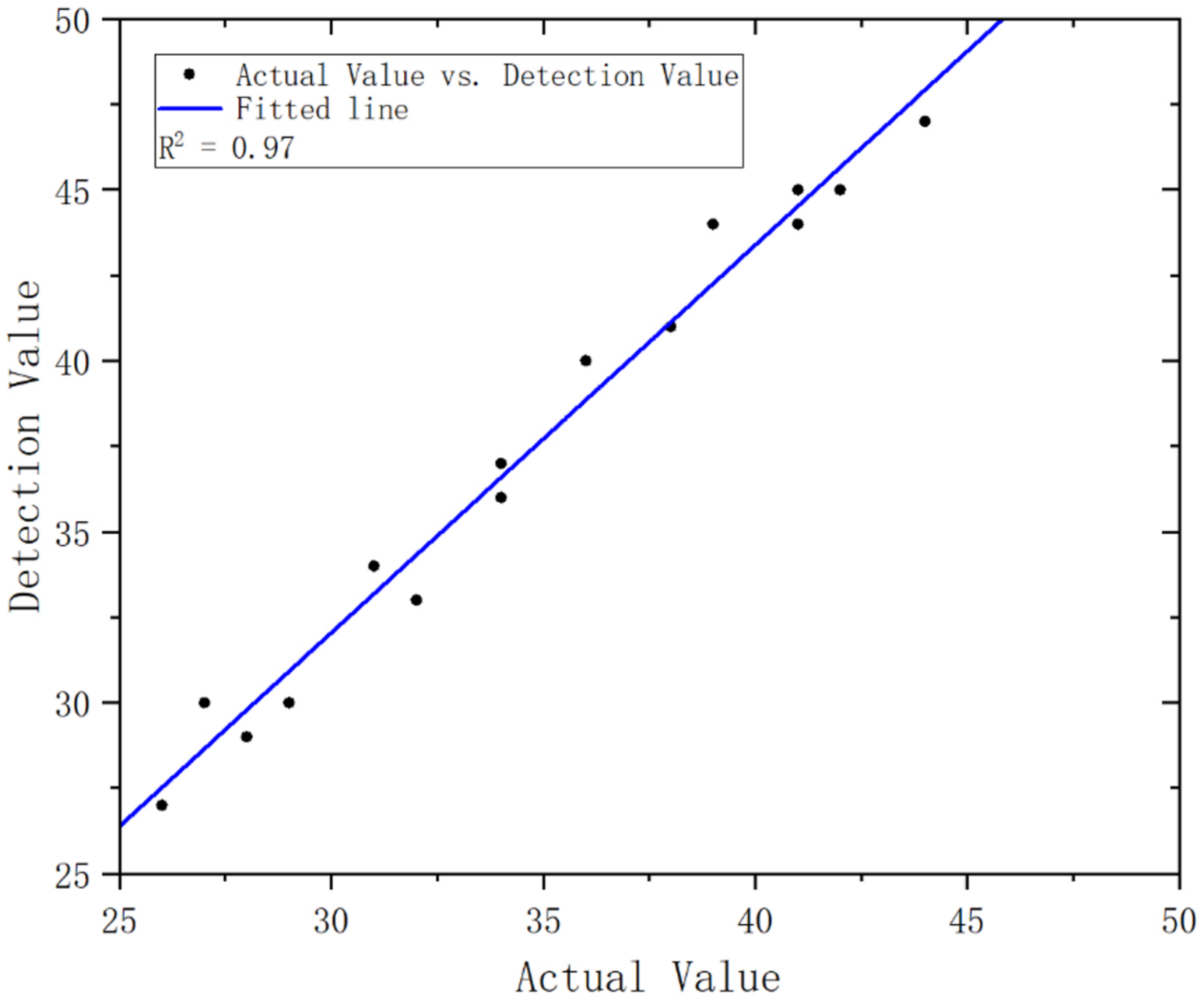

Supplement: Supplementary 1 — Figs. S1 to S6 [file plantphenomics.0258.f1.zip › figure S5.pdf]

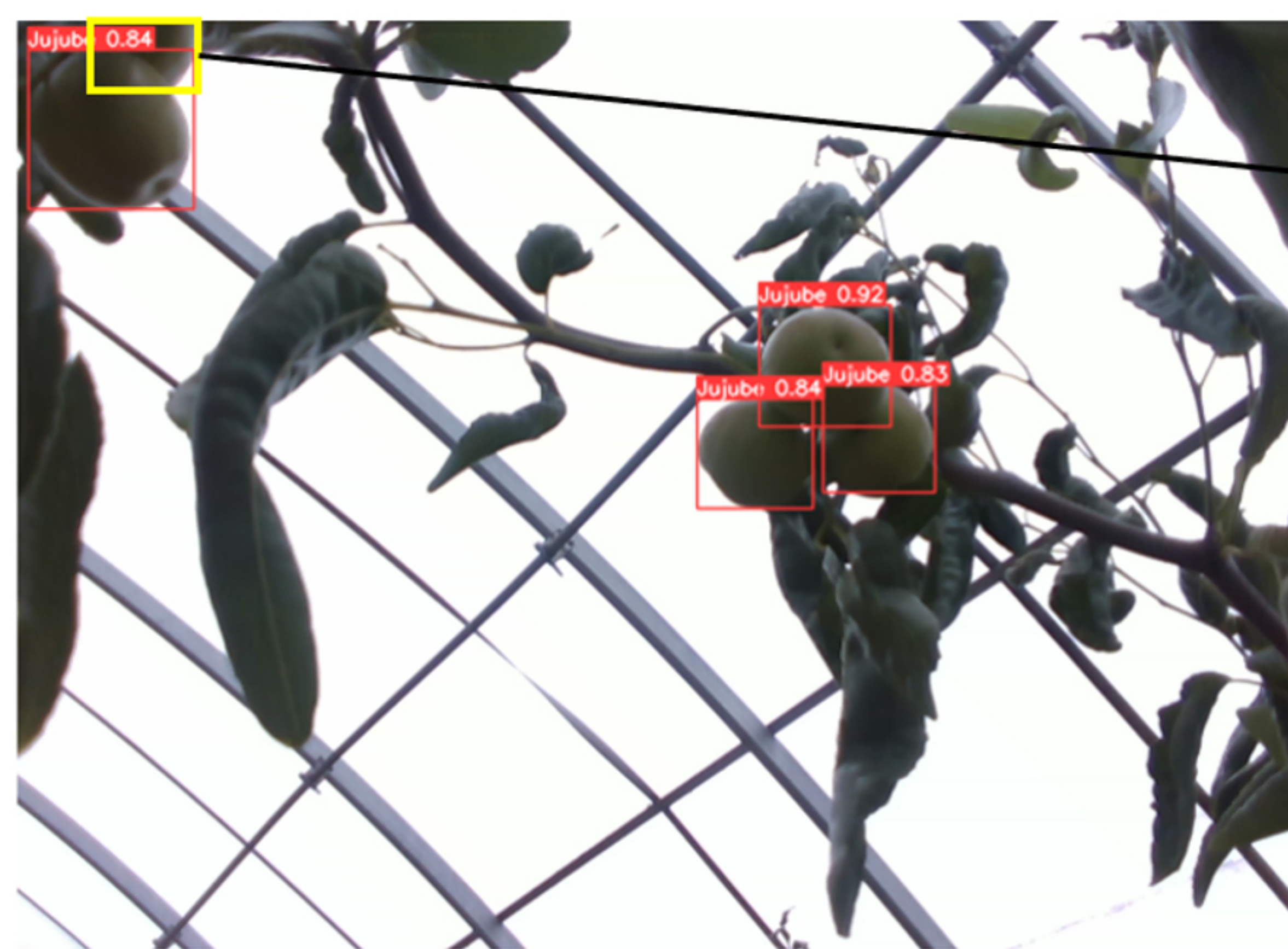

(a)

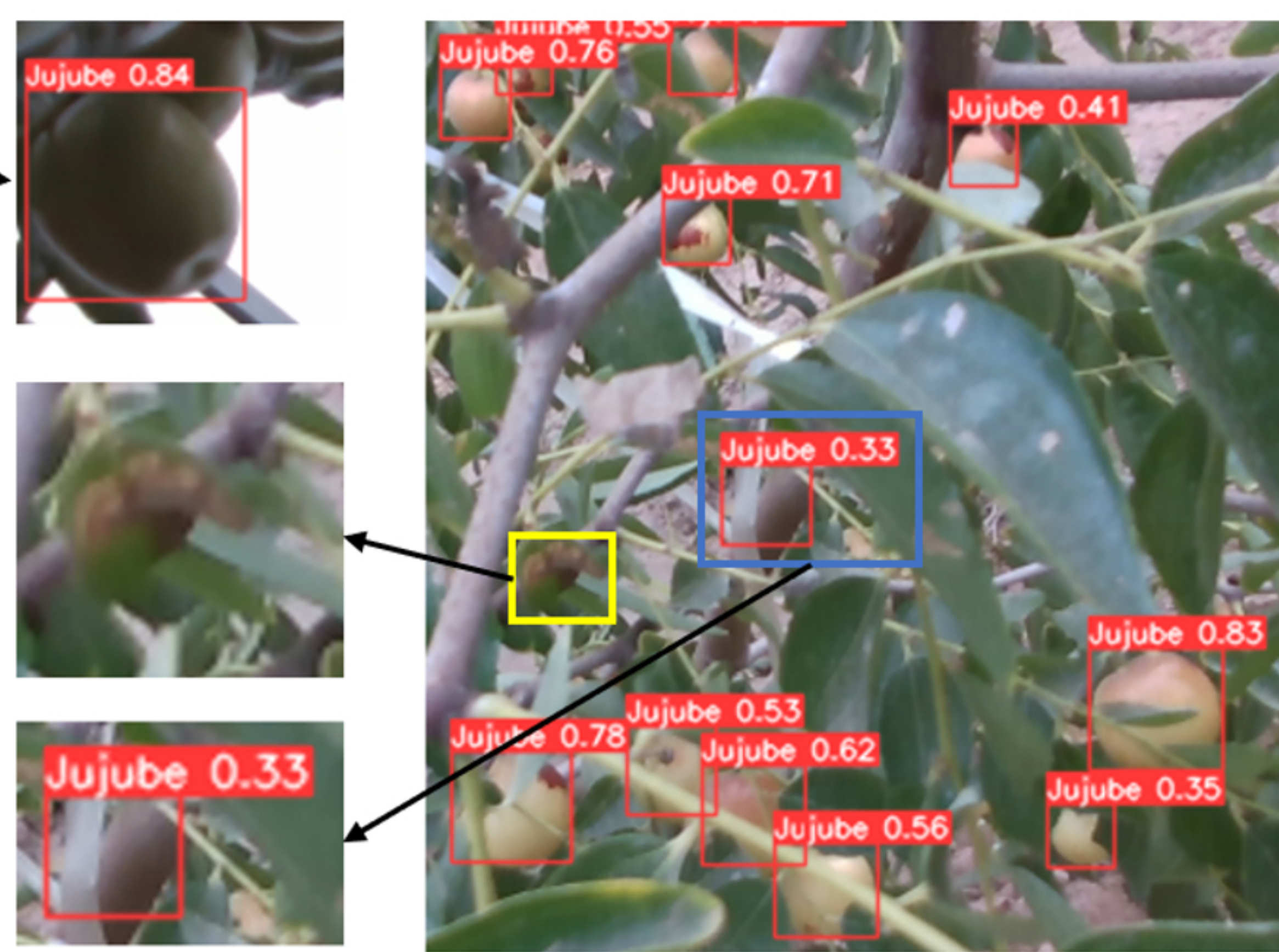

(b)

Supplement: Supplementary 1 — Figs. S1 to S6 [file plantphenomics.0258.f1.zip › figure S6.pdf]
